# Two bis-ligand-coordinated Zn(ii)-MOFs for luminescent sensing of ions, antibiotics and pesticides in aqueous solutions†

**DOI:** 10.1039/d2ra00376g

**Published:** 2022-03-09

**Authors:** Zhao-Di Zhou, Shi-Qi Li, Yin Liu, Bin Du, Yuan-Yue Shen, Bao-Yi Yu, Chong-Chen Wang

**Affiliations:** Key Laboratory of Urban Agriculture (North China), Ministry of Agriculture, College of Biological Sciences Engineering, Beijing University of Agriculture Beijing 102206 P.R. China yubaoyi123@hotmail.com; Beijing Key Laboratory of Functional Materials for Building Structure and Environment Remediation, Beijing University of Civil Engineering and Architecture Beijing 100044 P.R. China; Beijing Key Laboratory of Agricultural Product Detection and Control of Spoilage Organisms and Pesticide Residue, Faculty of Food Science and Engineering, Beijing University of Agriculture Beijing Beijing 102206 P.R. China

## Abstract

Two organometallic complexes with two and three-dimensional architectures were constructed by using multiple ligands and Zn(ii) ions: [Zn_3_(BTC)_2_(DTP)_4_(H_2_O)_2_]·(H_2_O)_4_ (Zn-1) (BTC = benzene-1,3,5-tricarboxylic acid and DTP = 3,5-di(1,2,4-triazol-1-yl)pyridine) and [Zn_2_(NTD)_2_(DTP)] (Zn-2) (NTD = 1,4-naphthalenedicarboxylic acid). The as-prepared complexes were characterized by single-crystal X-ray diffraction (SCXRD), elemental analysis, powder X-ray diffraction (PXRD), Fourier transform infrared spectroscopy (FT-IR), thermogravimetric analysis (TGA) and fluorescence analysis. Fluorescence sensing tests revealed that the two complexes are effective, sensitive and selective toward cationic Fe^3+^ and anionic MnO_4_^−^ and Cr_2_O_7_^2−^. During the antibiotic sensing process, cefixime (CFX) for Zn-1 and nitrofurantoin (NFT) for Zn-2 exhibited the highest quenching efficiencies. For sensing pesticides, the highest quenching efficiencies were exhibited by imidacloprid (IMI) toward Zn-1 and Zn-2. The fluorescence quenching of the complexes that was induced by antibiotics, pesticides and MnO_4_^−^ was attributed to both the inner filter effect (IFE) and the fluorescence resonance energy transfer (FRET) effect.

## Abstract 2

The fluorescence and pollutant sensing of two Zn-complexes was investigated, and the corresponding mechanism was proposed.

## Graphical abstract

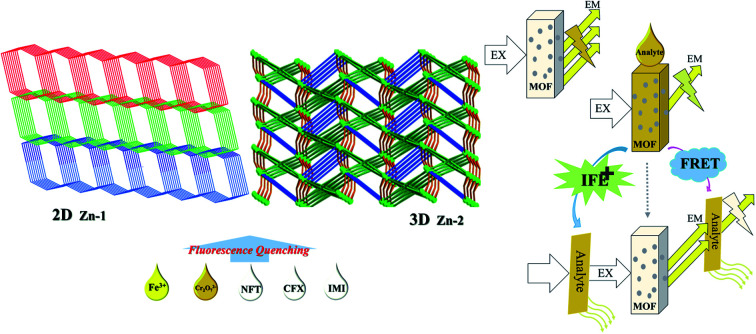

## Introduction

Currently, accompanying the enormous development of industry, agriculture and pharmaceuticals, environmental pollution, especially water pollution, has emerged as an increasingly severe issue in ecology and human health.^1,2^ Among the diverse inorganic pollutants, iron is a necessary element for the human body, and a higher dose uptake of Fe^3+^ from drinking water or food can cause severe health problems.^3^ Cr_2_O_7_^2−^ and MnO_4_^−^ anions are necessary strong oxidants that are widely used in laboratories, the manufacturing industry and chemical production, but these anions are nonbiodegradable, exhibit high toxicity and are known to be potent carcinogens.^4,5^

Antibiotics are vital drugs that are effective against and prevent bacterial infection.^6,7^ To date, hundreds of antibiotics have been commercialized and applied in human disease treatment, aquaculture and husbandry.^8–10^ Antibiotics are excessively abused in and discharged from livestock and poultry, and superfluous residuals overflow from aquaculture production.^11,12^ Occasionally, wastewater is directly discharged into or improperly treated in the aquatic system. All of these factors can cause severe antibiotic resistance and even ‘superbacteria’ breeding in the environment.^13,14^ Similarly, pesticide pollutants, which are another class of typical poisonous organic pollutants, attract tremendous attention in pollution monitoring.^15^ Although pesticides are widely used and greatly contribute to yield in modern agriculture, pesticide residues in agricultural production or in agricultural runoff and drainage are continuous hazards, which may threaten the environment and human health *via* direct digestion or bioaccumulation alongside food chains.^16^ Current detection methods are commonly based on expensive and complicated apparatuses these methods include ion mobility spectrometry, atomic absorption spectrometry (AAS), gas chromatography (GC), high-performance liquid chromatography (HPLC), and liquid chromatography-tandem mass spectrometry (LC-MS).^17–20^

Recently, metal–organic frameworks (MOFs), which are a novel type of porous crystalline material, have been self-assembled by using metal centers or metal clusters and organic linkers to construct periodic networks, and they have attracted tremendous research interest.^21–23^ MOFs demonstrate a broad range of unique properties, such as tunable porosity, adjustable architecture, large surface area, superstability, and various metal sources, which enable MOFs to be extensively explored and applied in gas storage,^24^ catalysis,^25^ drug delivery,^26^ magmatic materials,^27^ pollutant removal,^28^ and electronic and fluorescence sensing.^29^ Many studies have focused on luminescent metal–organic frameworks (LMOFs).^30,31^ As a subfamily of MOFs, LMOFs have been widely explored based on their prominent optical properties and used in many fields, such as the detection of metal cations,^32–35^ anions,^5,36,37^ solvents,^38,39^ volatiles,^40^ antibiotics,^41–45^ pesticides,^46–48^ nitro-containing compounds,^49–53^ pH,^54^ amino acids,^55^ and other organic molecules.^30,31^ During these processes, LMOFs displayed the great advantages of simplified equipment, fast response time, high selectivity and sensitivity, recyclability and ease of use.^31^ To date, fluorescence sensing progress with LMOFs has been due mainly to fluorescence turn-on,^33,56^ fluorescence turnoff or fluorescence shifts.^28,46,57^ Fluorescence turnoff, or fluorescence quenching, in most cases, is based on material transformation,^58,59^ electron transfer^57^ and/or energy competition/transfer mechanisms.^28,46^ Among the LMOFs, luminescence source emissions that are based on lanthanide cores are interesting due to the inherent narrow band luminescence, long lifetime, and large Stokes shift.^60^ In addition to lanthanide LMOFs, *d*-block LMOFs have been reported more recently as promising fluorescence sensors. Among them, *d*^10^ configuration metals, especially Zn^2+^-based LMOFs, have excellent fluorescence capabilities and are considered to be good choices as fluorescence sensing materials.^61–64^ A common LMOF is constructed from carboxylic groups containing ligands and/or N-donor ligands (pyridines or imidazole groups, *etc*).^65^ During a photoluminescent process, organic ligands often function as ‘antennae’ to absorb incoming light thereafter, the interaction with metal centers or other auxiliary ligands affects the direct intrinsic emission properties, thereby producing a distinct luminescence spectrum of the LMOF.^66^ The combination of the two types of ligands may enhance the advantages of both components and enrich the coordination environments of the center ions of the MOFs, thereby producing various novel topologies and fascinating fluorescence properties.^21^

Based on the above considerations, we devoted our efforts to the rational design of wonderful architectures of LMOF materials and explored the possible effects on their structural and fluorescence properties. In this context, two 2D and 3D Zn-based bisligand coordinated complexes were synthesized: Zn-1, [Zn_3_(BTC)_2_(DTP)_4_(H_2_O)_2_]·(H_2_O)_4_ (BTC = benzene-1,3,5-tricarboxylic acid and DTP = 3,5-di(1,2,4-triazol-1-yl)pyridine) and Zn-2, [Zn_2_(NTD)_2_(DTP)] (NTD = 1,4-naphthalenedicarboxylic acid). Furthermore, the structural information of the two complexes was evaluated by single-crystal X-ray diffraction (XRD), elemental analysis, powder X-ray diffraction (PXRD), infrared spectroscopy and thermogravimetric analysis (TGA). Their fluorescence properties and fluorescence sensing capabilities toward cations, anions, and selected antibiotics and pesticides were also explored. Fluorescence sensing tests revealed that the two complexes were effective, sensitive and selective toward Fe^3+^ among the cations and MnO_4_^−^ and Cr_2_O_7_^2−^ among the anions. During the antibiotic sensing process, cefixime (CFX) for Zn-1 and nitrofurantoin (NFT) for Zn-2 exhibited the highest quenching rates. For sensing pesticides, the highest quenching efficiencies were exhibited by imidacloprid (IMI) for both Zn-1 and Zn-2. The fluorescence quenching of the complexes that was induced by the ions, antibiotics and pesticides in this research was due to both the inner filter effect (IFE) and the fluorescence resonance energy transfer (FRET) effect.

## Experimental section

All the materials and solvents were obtained commercially and used without any further purification. ZnSO_4_·7H_2_O, benzene-1,3,5-tricarboxylic acid (BTC), 1,4-naphthalenedicarboxylic acid (NTD) and acetonitrile were supplied by Alfa Aesar Chemical Reagent Co. Ltd. 3,5-di(1,2,4-triazol-1-yl) pyridine (DTP) was synthesized following a reported method.^67^

Powder X-ray diffraction (PXRD) measurements were performed using a Bruker-avance X-ray diffractometer equipped with a Cu-target tube and a graphite monochromator scanning over the range of 5–50° at the rate of 0.2° s^−1^. The simulated X-ray diffraction patterns were generated from proper treated Cif files of the related complexes crystals by using the Mercury software. A model METTLER TOLEDO 1600 TH thermal analyzer was used to record TG curves at a heating rate of 10 °C min^−1^ over the temperature ranging from r. t. to 800 °C in a flowing nitrogen atmosphere of 10 mL min^−1^ using platinum crucibles. Elemental analyses for C, H, and N were carried out using a PerkinElmer 240 CHN elemental analyzer. The Fourier transform infrared (FT-IR) spectra were obtained using an Agilent Cary630 spectrophotometer in the range of 4000 to 500 cm^−1^. UV-vis spectroscopic studies were carried out using a Varian UV50 Conc spectrophotometer. All luminescence measurements were performed using an Agilent Cary Eclipse fluorescence spectrophotometer at r. t.

### Single crystal X-ray diffraction

Single crystals suitable for X-ray diffraction analysis of the complexes Zn-1 and Zn-2 were grown from the synthesis progresses. During the measurement, crystals were selected and mounted on a Bruker APEX-II CCD diffractometer and were kept at 150.0(1) K during data collection using a Mo-K*α* radiation (*λ* = 0.71073 Å). Integration and scaling of intensity data was performed by using the SAINT program.^68^ Data were corrected for the effects of absorption using SADABS.^69,70^ Using Olex2,^71^ the structures were solved with the SIR 2004 structure solution program^72^ using Direct Methods. Further, the structures were refined with the ShelXL^73^ refinement package using Least Squares minimization. Non-hydrogen atoms were anisotropically refined and the hydrogen atoms in the riding mode^71,74^ and isotropic temperature factors fixed at 1.2 times U(eq) of the parent atoms (1.5 times for methyl groups).

A summary of the crystallographic data and refinement parameters is provided in Table S1 (see the ESI†).

### Syntheses of Zn-1 and Zn-2

Into a Teflon-lined stainless-steel autoclave (25 mL), ZnSO_4_·7H_2_O (2 eq., 0.0135 g, 0.047 mmol), a carboxylic group-bearing ligand (1 eq., 0.024 mmol), DTP (1 eq., 0.0050 g, 0.024 mmol), KOH (0.5 eq., 0.012 mmol, 0.0006 g) and suitable solvents were added and sealed. Then, the autoclave was maintained at 80 °C for 72 h. Thereafter, the reaction was cooled to r. t. The crystallized solid material was filtered and washed with deionized water. The white crystallized solid materials were dried in open air at r. t. Crystals that were suitable for X-ray diffraction analysis were obtained from the synthesis process and analyzed without further treatment.

For Zn-1, BTC (0.0029 g) and water (5 mL) were used (0.0085 g, yield: 68.9%). Anal. elemental analysis calculated for C_54_H_46_N_28_O_18_Zn_3_ (wt%): C, 41.28% H, 2.95% N, 24.96% found: C, 41.68% H, 2.82% N, 24.82% IR (neat, *ν* cm^−1^): 3114(bs), 1612(s), 1560(s), 1418(s), 1346(s), 1278(s), 1210(s), 1233(s), 990(s), 973(s), 893(s), 788(s), 751(s), 732(s), 691(s), 667(s), 639 (s), 537(s).

For Zn-2, 1,4-naphthalenedicarboxylic acid (NTD) (0.0051 g) and acetonitrile (2.5 mL), water (2.5 mL) were used (0.0063 g, yield: 60%). Anal. elemental analysis (EA) for C_33_H_19_N_7_O_8_Zn_2_ (wt%): C, 51.32% H, 2.48% N, 12.70% found: C, 51.52% H, 2.67% N, 12.83% IR (neat, *ν* cm^−1^): 3084(bs), 1585(s), 1600(s), 1534(s), 1467(s), 1411(s), 1353(s), 1262(s), 1213(s), 1137(s), 975(s), 904(s), 848(s), 788(s), 686(s), 669(s), 641(s), 576(s).

### Analyte quenching test

In a typical experiment, a stock suspension of finely ground Zn-complex powder (5 mg) in H_2_O (25 mL) was sonicated for 15 min, and aqueous solutions of various analytes (10 mL) were prepared. During the photoluminescence test, the Zn-containing compound suspension (1.5 mL) and an analyte solution (1.5 mL) were well mixed and added to a cuvette at r. t. Thereafter, the luminescent spectra of the suspensions were evaluated under excitation at various wavelengths (278 nm for Zn-1 and 259 nm for Zn-2).

The used analytes include the following: cations (2 mM): MCl_1-3_ (M = K^+^, Na^+^, Mg^2+^, Ca^2+^, Ni^2+^, Co^2+^, Mn^2+^, Cu^2+^, Fe^2+^, Zn^2+^, Cd^2+^, Pb^2+^, Ba^2+^, Al^3+^, Cr^3+^, La^3+^ and Fe^3+^) anions (2 mM): K_1-2_X (X = F^−^, Cl^−^, Br^−^, I^−^, Ac^−^, SCN^−^, NO_3_^−^, ClO_3_^−^, ClO_4_^−^, MnO_4_^−^, HPO_4_^2−^, H_2_PO_4_^−^, CO_3_^2−^, B_4_O_7_^2−^, SO_3_^2−^, SO_4_^2−^ and Cr_2_O_7_^2−^) antibiotics (0.2 mM): lactams (penicillin V potassium, PVK; benzylpenicillin potassium, PK; amoxicillin, AML; cefixime, CFX), aminoglycosides (gentamicin, GTM; kanamycin KNM; tobramycin, TOB; streptomycin, SM), chloramphenicols (chloramphenicol, CAP; thiamphenicol, TAP), macrolides (roxithromycin, ROX; azithromycin, AZM), nitrofurans (nitrofurazone, NFZ; nitrofurantoin, NFT), nitroimidazoles (metronidazole, MNZ; 1,2-dimethyl-5-nitroimidazole, DMZ) and sulfonamides (sulfamethazine, SMZ) and pesticides (0.2 mM): (dipterex, DIP; pentachloro-nitrobenzene, PCNB; imazalil, IMZ; glyphosate, GLY; chlorothalonil, TPN; carbendazim, CAR; 2.4-dichlorophenoxyacetic acid, 2,4-D; imidacloprid, IMI; metamitron, MMT; thiophanate-methyl, TPM and nitenpyram, NTP).

### Luminescence kinetic titration

For the ions and the pesticides, in a typical experiment, an aqueous suspension of Zn complex was prepared by the scattering addition of 4 mg of well mashed complexes into distilled water (40 mL). Thereafter, the suspension was kept under ultrasonic conditions for 30 min. Afterward, 2 to 10 μL aliquots from the analyte's stock solution were injected and vortexed with the aqueous suspension (4 mL). Then, the luminescence spectra were recorded. The analytes' stock solutions were prepared in two concentrations: 20 mM for ions and 5 mM for pesticides.

For the antibiotics (CFX and NFT), a series of antibiotic solutions at various concentrations (0, 0.001, 0.005, 0.01, 0.02, 0.04, 0.05, 0.06, 0.08, 0.09, 0.1, 0.2, 0.3, 0.4, 0.5 and 0.6 mM) were prepared. Luminescence measurements were performed by the addition and stirring of every analyte's solution (1.5 mL) with a 0.2 mg mL^−1^ Zn-complex suspension (1.5 mL).

### Anti interference experiments

Stock solutions containing the cations (2 mM) (K^+^, Na^+^, Mg^2+^, Ca^2+^, Ni^2+^, Co^2+^, Mn^2+^, Cu^2+^, Zn^2+^, Cd^2+^, Pb^2+^, Ba^2+^, In^3+^, Al^3+^ and Cr^3^), the anions (2 mM) (F^−^, Cl^−^, Br^−^, I^−^, Ac^−^, SCN^−^, NO_3_^−^, ClO_3_^−^, ClO_4_^−^, HPO_4_^2−^, H_2_PO_4_^−^, CO_3_^2−^, B_4_O_7_^2−^, SO_3_^2−^ and SO_4_^2−^), Fe^3+^ (4 mM), MnO_4_^−^ (4 mM), Cr_2_O_7_^2−^ (4 mM) were prepared and stock suspensions of the complex with the concentrations of 0.2 mg mL^−1^ and 0.4 mg mL^−1^ were obtained before the fluorescence analysis. The fluorescence intensity of the sensor without or with the presence of interfering ions was analyzed with a mixture that was obtained from the injection of 1.5 mL water or the solution with the interfering ions to 1.5 mL sensor's suspension (0.2 mg mL^−1^). Finally, the fluorescence intensity of the sensor together with the interfering ions and the quencher was mixed from 1.5 mL the solution with the interfering ions, 0.75 mL sensor's suspension (0.4 mg mL^−1^) and 0.75 mL quencher's solution (4 mM).

### Recyclability of luminescence experiments

After fluorescence detection analysis, the Zn-based complex was centrifuged and washed repeatedly with deionized water. Afterward, the solid sediment was collected and used in the next fluorescence detection test. This procedure was repeated for an additional 4 cycles.

## Results and discussion

### Structural characterization of Zn-MOFs

The structures of Zn-1 and Zn-2 were determined by X-ray single-crystal diffraction analysis (Table S1†). The results show that Zn-1 crystallized in the triclinic space group *P*1̄ (no. 2) and Zn-2 crystallized in the orthorhombic space *P*2_1_2_1_2_1_ (no. 19), respectively. Selected bond lengths and bond angles for Zn-1 and Zn-2 are listed in Tables S2 and S3.†

**Fig. 1:**
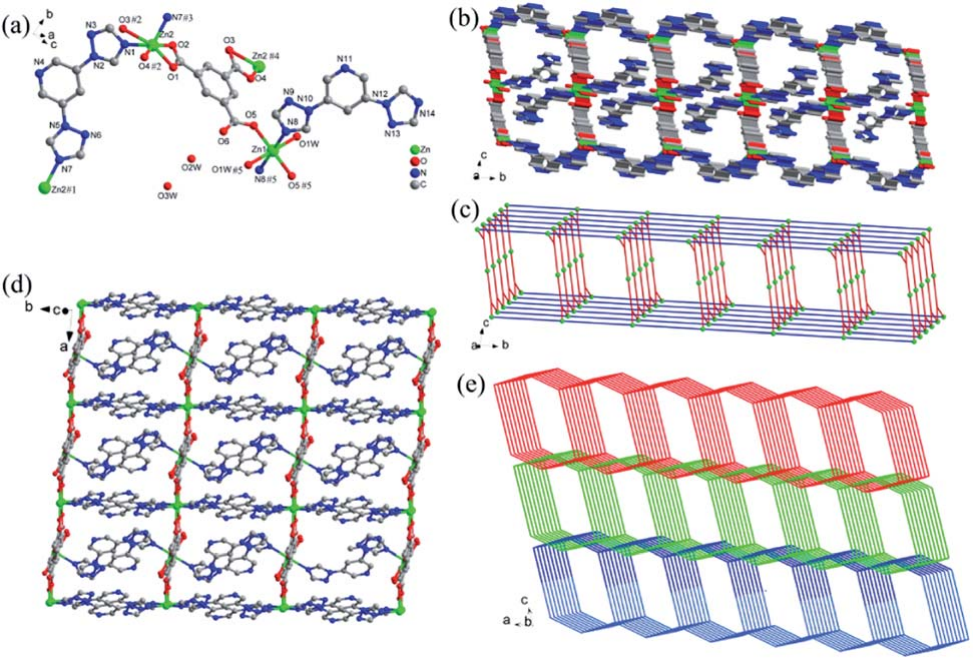
(a) The coordination environment of Zn(ii) ions and the ligands in Zn-1 (the hydrogen atoms and the disorder of the triazolyl moieties in the DTP ligand are omitted for clarity); (b) a view of the 2D metal layer along the *a*-axis; (c) a topological view of the 3,4-c net with the {6^3^}{6^5^ · 8} topology; (d) a view of the 2D metal layer along the *c*-axis; (e) the 2D layers interconnect to form a twofold interpenetrating 3D framework of Zn-1.

Fig. 1a shows that the asymmetric unit of Zn-1 includes one and a half Zn(ii) ions, one coordinated and two lattice water molecules, two DTP ligands and one fully deprotonated BTC^3−^ ligand. The Zn(ii)1 ion is six-ligated with two carboxylic oxygen atoms from BTC^2−^ ligands, two oxygen atoms from coordinated water molecules and another two nitrogen atoms from DTP ligands. The coordination environment enables Zn(ii)1 to form an octahedral coordination geometry. The Zn(ii)2 ion is four-coordinated with a tetrahedral geometry that is constructed by two oxygen atoms from two carboxylic groups in the BTC^2−^ ligands and two nitrogen atoms from two DTP ligands. The DTP ligands adopt two coordination modes: as a terminal ligand that is coordinated with a Zn(ii)1 ion and as a bridge between two Zn(ii)2 ions to form a 1D chain alongside the *b*-axis (Fig. 1b and d). The BTC^2−^ ligand, which adopts a μ_3_-η_1_: η_1_: η_1_ coordination mode, links one Zn(ii)1 and two Zn(ii)2 ions to generate a ladder-like 1D chain along the *a*-axis. Finally, an interconnected double-layer 2D network is created parallel to the *ab*-plane (Fig. 1c). Zn-1 exhibits large channels that run along the *a*-axis and *b*-axis. To reduce the pore voids, a 2-fold interpenetrating network is fabricated (Fig. 1e). The 2D network can be simplified as a 2-nodal (3,4)-connected new topology with the point symbol {6^3^}{6^5^ · 8}.^75^

**Fig. 2:**
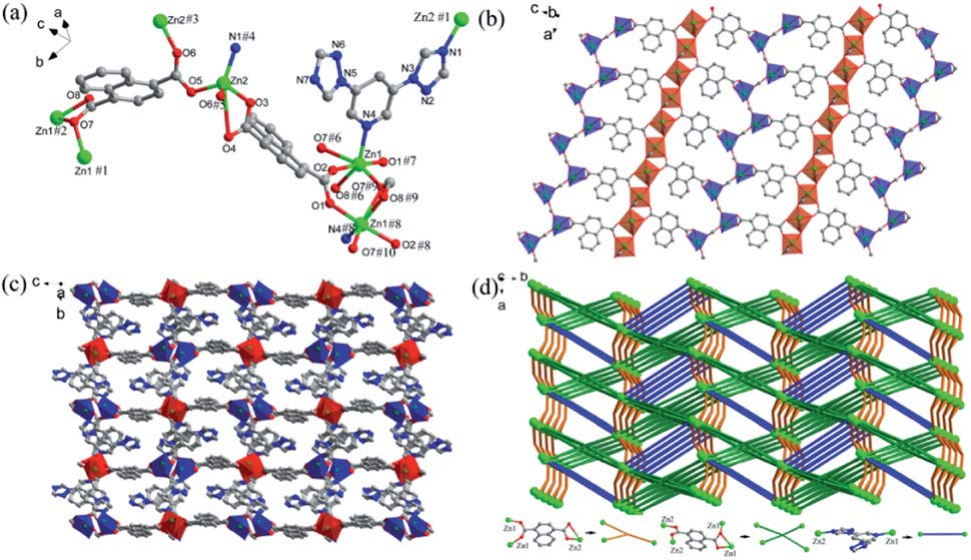
(a) The coordination environment of Zn(ii) ions and the ligands in Zn-2; (b) a view of the 2D metal layer along the *c* axis; (c) a perspective view of the 3D framework along the *a* axis; (d) a topological view of the 3,4,4,5-c net with a {4·5^3^·7^2^}{4·7^2^}{4^2^·5^5^·7^3^}{5^3^·7^2^·8} topology.

Fig. 2a exhibits the asymmetric unit of Zn-2 with two Zn(ii) ions, one DTP and fully deprotonated NTD^2−^ ligands. As shown in the figure, the Zn(ii)1 ion possesses a deformed tetragonal bipyramidal geometry (red polyhedron) in which the center ion is ligated with five carboxylic oxygen atoms from three NTD^2−^ ligands and one pyridyl nitrogen atom from DTP ligand. The Zn(ii)2 ion adopts a four-coordination to form a distorted tetrahedral configuration (blue polyhedron) that is constructed by three oxygen atoms from three NTD^2−^ ligands and one nitrogen atom from the triazolyl moiety of the DTP ligand. The adjacent Zn(ii)1 ions are linked together by two carboxylate groups from NTD^2−^ ligands to form infinite 1D chains that run along the *a*-axis with a Zn⋯Zn distance of 3.934(4) Å. Nevertheless, the nearby Zn(ii)2 ions are bridged together by one carboxylic group from the NTD^2−^ ligand to generate paddle wheel-like 1D chains that are also directed toward the *a*-axis (Fig. 2b and c). The two crystallographically independent NTD^2−^ ligands adopt different coordination modes: one NTD^2−^ ligand connects two Zn(ii)1 and two Zn(ii)2 ions along the *b*-axis to form the (*κ*^1^–*κ*^2^)–(*κ*^1^–*κ*^1^)-*μ*_4_ coordination mode the other NTD^2−^ ligand links two Zn(ii)1 ions and one Zn(ii)2 ion oriented toward the *c*-axis in the (*κ*^1^–κ^1^)–(κ^1^–κ^0^)-*μ*_3_ bridging mode. Next, the DTP ligand bridges two Zn(ii) ions (Zn(ii)1 and Zn(ii)2). In the direction of the *a*-axis, the DTP and NTD^2−^ ligands alternatively connect the two metal chains. Finally, the metal chains and the NTD^2−^ and DTP linkers build a 3D framework (Fig. 2d). Topologically, Zn-2 can be viewed as a 4-nodal (3,4,4,5)-connected new topology with the point symbol {4·5^3^·7^2^}{4·7^2^}{4^2^·5^5^·7^3^}{5^3^·7^2^·8}.^75^

### FT-IR, PXRD and thermogravimetric analysis

The FT-IR spectra (Fig. S1†) of Zn-1 and Zn-2 were measured, and the obtained curves are displayed in Fig. S1.† The characteristic stretching vibration of C=O in the protonated carboxy groups in the H_2_NTD and H_3_BTC ligands was located at approximately 1680 cm^−1^, which completely disappeared from the profiles of the metal-containing compounds Zn-1 and Zn-2. Instead, new peaks appeared at 1610 and 1350 cm^−1^ for Zn-1 and 1589 and 1350 cm^−1^ for Zn-2, which should be ascribed to asymmetric and symmetric stretching vibrations of C=O in the Zn(ii)-coordinated carbonyl groups of the ligands.

**Fig. 3:**
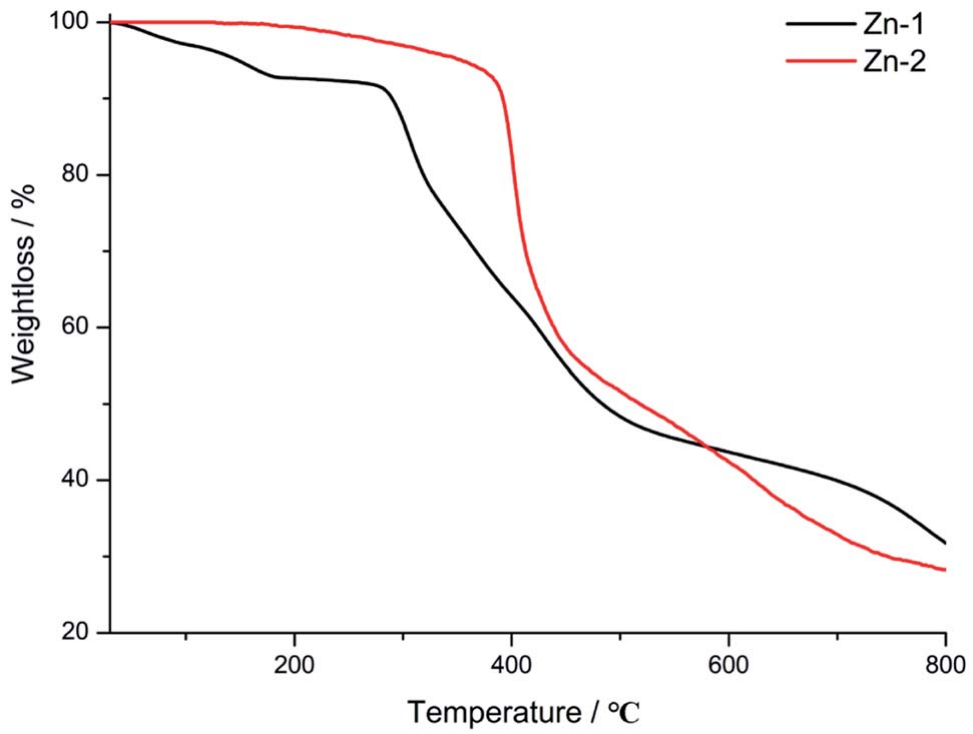
Thermogravimetric curves of Zn-1 and Zn-2 in the temperature range of 30–800 °C under a N_2_ atmosphere.

To identify the phase purities of Zn-1 and Zn-2 compounds before photoluminescence measurements, PXRD was performed. As depicted in Fig. S2,† the peaks of the experimental plots match well with those of the simulated plots, thereby suggesting that the bulk synthesis of the compounds has high phase purity. To evaluate the thermal stabilities of the compounds, thermogravimetric analyses of the compounds were carried out under a N_2_ atmosphere in the temperature range from r. t. to 800 °C (10 °C min^−1^). As shown in Fig. 3, from r. t. to 182 °C, a first weight losses of Zn-1 was and 7.2% which correspond to the loss of coordination and lattice water molecules (calcd.: 5.2% and 6.9%). At 280 °C, sharp weight loss occurred due to structural decomposition for Zn-1. The next, the guest-free framework Zn-2 exhibited satisfactory thermal stability and remained intact up to 380 °C, after that, the compound began to suffer rapid mass loss. In addition, the stability of the two MOFs also was explored in different pH solution in a range from 2 to 12. Fig. S3† showed that the phase purity of the two complexes was consistence with the ones that simulated from SCRD data, indicating strong stability of the two complexes under the test condition.

### Fluorescence

**Fig. 4:**
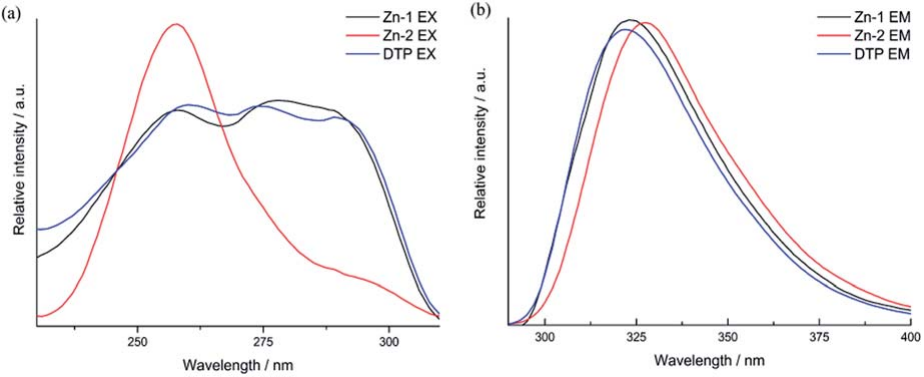
Solid-state fluorescence spectra of Zn-1 and Zn-2 and the free ligand at r. t.: excitation (a) and emission (b).

The conjugation of *d*^10^-metals with N or carboxylic groups that contain organic linkers usually generates complexes that exhibit excellent luminescence properties. The solid-state luminescent capabilities of compounds Zn-1 and Zn-2, together with the free ligand DTP, were investigated at r. t. (Fig. 4). Overall, all the two complexes and DTP ligand show a singlet apex profile and display maximum emission at wavelengths around 322 nm (Fig. 4b). The emission could be attributed to the typical electronic transitions of intraligand π → π* and *n* → π* orbitals based on emission of DTP ligand within the structures. However, the two complexes performed differently in excitation profiles, Zn-1 displays triplet apex spectra of excitation that are similar to those of DTP ligands, and Zn-2 exhibits singlet apex spectra of excitation at 258 nm.

To evaluate the sensing capabilities of Zn-complexes, grounded Zn-MOF samples (1 mg) were dispersed in 5 mL of H_2_O, ultrasonicated for 10 min, and left to rest for an additional 2 hours. For metal ions, concentrations of 2 mM (MCl*x*: M = K^+^, Na^+^, Mg^2+^, Ca^2+^, Ni^2+^, Co^2+^, Mn^2+^, Cu^2+^, Zn^2+^, Cd^2+^, Pb^2+^, Ba^2+^, Fe^2+^, In^3+^, Al^3+^, Cr^3+^, La^3+^ and Fe^3+^) or 2 mM (K_*y*_X: X = F^−^, Cl^−^, Br^−^, I^−^, Ac^−^, SCN^−^, NO_3_^−^, ClO_3_^−^, ClO_4_^−^, MnO_4_^−^, HPO_4_^2−^, H_2_PO_4_^−^, CO_3_^2−^, B_4_O_7_^2−^, SO_3_^2−^, SO_4_^2−^ and Cr_2_O_7_^2−^) were prepared. The fluorescence quenching effects of these cations and anions were induced by the addition of the analyte solution (1.5 mL) into a complex suspension (1.5 mL). Finally, the resulting mixed suspension was subjected to fluorescence measurement, and the concentrations of the analyte and metal complex in the final mixture were 1 mM and 0.1 mg mL^−1^, respectively.

**Fig. 5:**
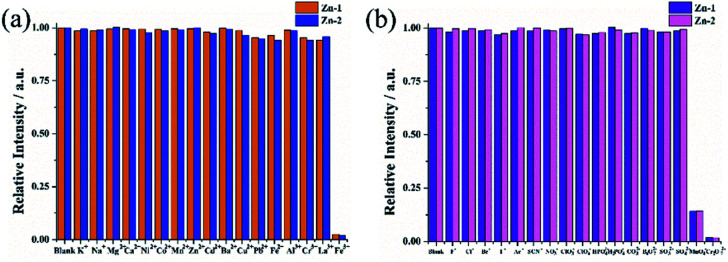
Quenching efficiencies of Zn-1 and Zn-2 dispersed in aqueous solutions those were treated with various cations (a) and anions (b) (1 mM).

As shown in Fig. 5a and b, except for Fe^3+^, Cr_2_O_7_^2−^ and MnO_4_^−^, all selected cations and anions showed negligible enhancement or quenching of the fluorescence intensity of the Zn complexes. For the cations, the introduction of Fe^3+^ into the suspension of Zn complexes caused a dramatic quenching effect, and the quenching rates (1 − *I*/*I*_0_) were 97.6% and 98.0% for Zn-1 and Zn-2, respectively. For the anions, Cr_2_O_7_^2−^ and MnO_4_^−^ exhibited obviously drastic turn-off quenching effects toward the Zn complexes. The initial fluorescence intensity quenching rates were 85.8% and 85.7% when MnO_4_^−^ was used with the two Zn complexes, and the quenching rates reached 98.2% and 98.3% when Cr_2_O_7_^2−^ was employed.

The above experiment was performed at a singlet concentration. Next, titration experiments were executed to establish the relationship between the quencher concentration and fluorescence intensity to evaluate the interior competence. In addition, the quenching constant *K*_sv_ was calculated *via* the Stern–Volmer (SV) equation: *I*_0_/*I* − 1 = *K*_sv_[*C*], where [*C*] is the molar concentration of the analyte and *I*_0_ and *I* represent the luminescence intensity of the Zn complex in H_2_O in the presence and absence of analytes, respectively.

As shown in Fig. S4–S9,† as the ions were gradually added, the fluorescence intensities of all the tested Zn-complexes decreased proportionally. The plots exhibit satisfactory linear correlation in low analyte concentration ranges (0–0.1 mM for Fe^3+^ and Cr_2_O_7_^2−^, 0–0.35 mM for MnO_4_^−^), while as the concentration of the analytes increases, the curves deviate upwardly from linearity. The *K*_sv_ values (Table S4†) were calculated to be 8.03 × 10^3^ and 1.53 × 10^4^ M^−1^ when Fe^3+^ was used for Zn-1 and Zn-2, respectively. For sensing the anions, the *K*_sv_ values are 2.40 × 10^3^ and 2.78 × 10^3^ M^−1^ (MnO_4_^−^) and 1.57 × 10^4^ and 1.73 × 10^4^ M^−1^ (Cr_2_O_7_^2−^) for the two Zn complexes. In addition to the SV equation, the limit of detection (LOD) is another useful tool for evaluating the sensing capabilities of fluorescence sensors, which is defined as LOD = 3*σ*/*K*_sv_ (*σ* is the relative standard error that is calculated from ten repeated blank measurements). For evaluating the sensing capabilities of Zn-1 and Zn-2, the LOD values are 9.52 × 10^−7^ and 4.99 × 10^−7^ M for detecting Fe^3+^ 3.19 × 10^−6^ and 2.75 × 10^−6^ M for detecting MnO_4_^−^ and 4.89 × 10^−7^ and 4.43 × 10^−7^ M for detecting Cr_2_O_7_^2−^.

**Fig. 6:**
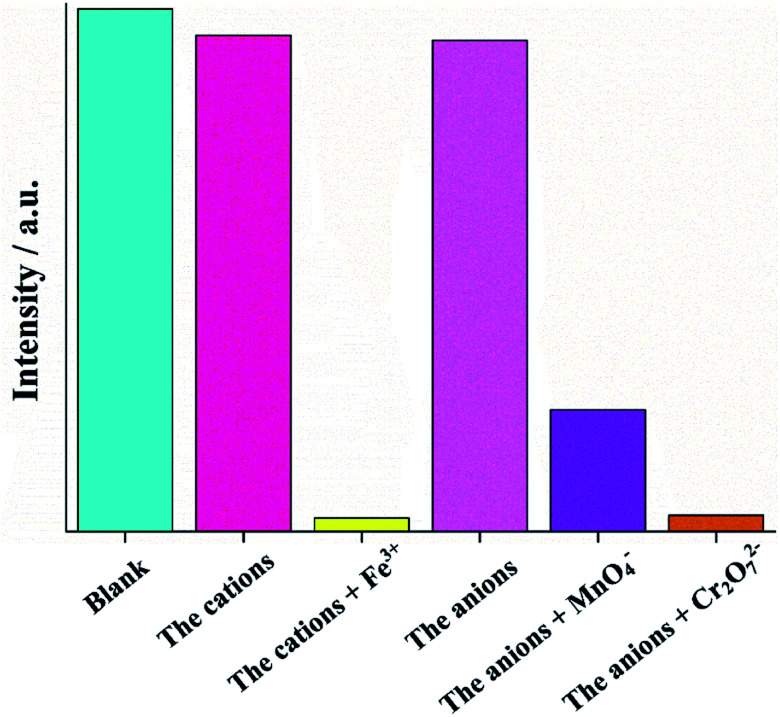
Comparison of fluorescence intensity before and after the addition of Fe^3+^ and Cr_2_O_7_^2−^ to the suspension of Zn-2 with various cations and anions (cations = K^+^, Na^+^, Mg^2+^, Ca^2+^, Ni^2+^, Co^2+^, Mn^2+^, Cu^2+^, Zn^2+^, Cd^2+^, Pb^2+^, Ba^2+^, In^3+^, Al^3+^ and Cr^3+^ anions = F^−^, Cl^−^, Br^−^, I^−^, Ac^−^, SCN^−^, NO_3_^−^, ClO_3_^−^, ClO_4_^−^, HPO_4_^2−^, H_2_PO_4_^−^, CO_3_^2−^, B_4_O_7_^2−^, SO_3_^2−^ and SO_4_^2−^).

The selectivities of Zn-1 and Zn-2 for the quenchers can be assessed by anti-interference experiments. Herein, Zn-2 was selected as an example, as shown in Fig. 6, in the absence of Fe^3+^ ions, the presence of other cations caused no obvious decrease in the initial fluorescence intensity of Zn-2, and fluorescence quenching was readily observed after the addition of Fe^3+^ ions. Similarly, none of the other anions obviously decreased the fluorescence intensity of Zn-2 in the absence of MnO_4_^−^ and Cr_2_O_7_^2−^, while fluorescence quenching was readily observed after the addition of MnO_4_^−^ or Cr_2_O_7_^2−^ ions.

### Antibiotic sensing

In addition to ions, common organic pollutants, such as antibiotics and pesticides, were also investigated in this research. For antibiotic and pesticide sensing, analytes at a concentration of 0.2 mM were prepared with the following antibiotics: lactams (PVK, PK, AML, CFX), aminoglycosides (GTM, KNM, TOB and SM), chloramphenicols (CAP and TAP), macrolides (ROX and AZM), nitrofurans (NFZ and NFT), nitroimidazoles (MNZ and DMZ) and sulfonamides (SMZ). The structural formulas of antibiotics is shown in Table S5.†

**Fig. 7:**
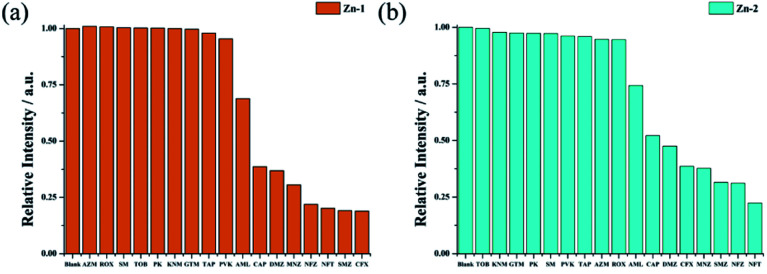
Quenching efficiencies of Zn-1 (a) and Zn-2 (b) dispersed in aqueous solutions that were treated with various antibiotics (0.1 mM).

During the sensing process, equal volumes of the analyte solution and Zn-complex suspension were mixed at a final concentration of 0.1 mM: 0.1 mg [M] mL^−1^. Overall, antibiotics TOB, KNM, GTM, PK, PVK, SM, TAP, ROX, and AZM did not contribute significant quenching for any of the two Zn complexes at an analyte concentration of 0.1 mM. Because these analytes significantly impact the emitter fluorescence, the two Zn complexes performed differently toward the different antibiotics. As shown in Fig. 7, in sensing the antibiotics, for Zn-1, CFX gives rise to the highest quenching rate, and the quenching rates are ranked as CFX > SMZ > NFT > NFZ > MNZ > DMZ > CAP > AML. Zn-2 performed differently from Zn-1, and the quenching rates for Zn-2 were in the order of NFT > NFZ ≈ SMZ > MNZ ≈ CFX > DMZ > CAP > AML.

To quantitatively evaluate the effects of the antibiotics on the sensing capability of the Zn complexes, titration experiments were performed by gradual addition of antibiotics into a suspension of the Zn complexes. The antibiotics that performed best in the singlet quenching test for each Zn complex were selected for the titration experiments (CFX for Zn-1 and NFT for Zn-2). As shown in Fig. S10 and S11,† the fluorescence intensity of the Zn complexes dropped gradually with increasing concentrations of the tested antibiotics. The *K*_sv_ values for CFX in suspensions of Zn-1 and NFT in Zn-2 were linearly correlated (*R*^2^ of 0.99) with the analyte number in low concentration ranges of 0–0.05 mM. The linearity changed to an upward curve when in a higher analyte concentration range. The obtained *K*_sv_ and LOD values for CFX are 2.94 × 10^4^ M^−1^ and 2.60 × 10^−7^ M with Zn-1. For NFT, they are 2.64 × 10^4^ M^−1^ and 2.90 × 10^−7^ M with Zn-2. The *K*_SV_ and LODs values of MOF based luminescent probes recently reported for sensing NFT and CFX are summarized in Table S7.†

### Pesticide sensing

**Fig. 8:**
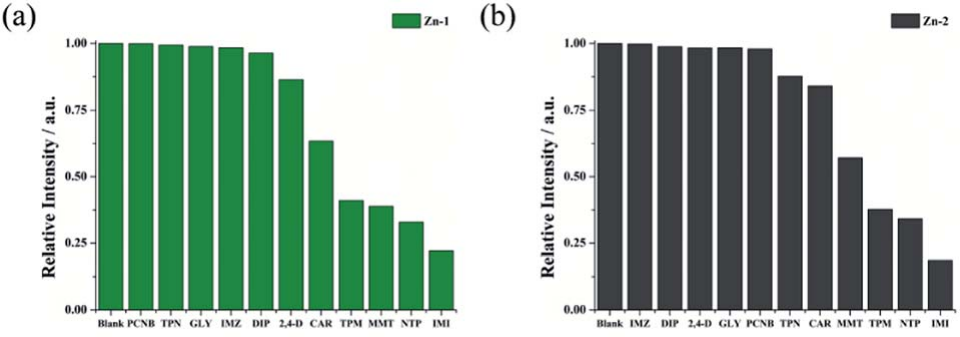
Quenching efficiencies of Zn-1 (a) and Zn-2 (b) dispersed in aqueous solutions and treated with various pesticides (0.1 mM).

The notable fluorescence sensing capabilities of the Zn complexes motivated us to expand their use in pesticide detection. Pesticides (DIP, PCNB, IMZ, GLY, TPN, CAR, 2,4-D, IMI, MMT, TPM and NTP) in H_2_O solution were prepared for sensing tests. The structural formulas of pesticides is shown in Table S6.† The experimental procedures that were applied for sensing pesticides resemble those used for antibiotics. Fig. 8 shows that four (PCNB, GLY, IMZ, and DIP) of the eleven pesticides have negligible effects on the fluorescence quenching of the two Zn complexes. The results revealed that IMI caused the highest quenching rate when Zn-1 was used then, the pesticides NTP, MMT and TPM gave rise to similar quenching efficiencies, and eventually, less significant quenching was observed for CAR and 2,4-D. When Zn-2 was employed, IMI also resulted in the highest quenching rate, followed by NTP and TPM. Finally, MMT, CAR and TPN caused moderate to less significant effects on fluorescence quenching.

Furthermore, pesticides IMI for Zn-1 and Zn-2 were selected in kinetic titration experiments. Obvious quenching of the luminescence intensity was observed as the pesticide concentrations gradually increased. As shown in Fig. S12 and S13,† the relative fluorescence intensity and the concentration of the analytes exhibit a well-fitted linear correlation (*R*^2^ = 0.99) in the low concentration 0–0.05 mM range, and the plots were upwardly curved for many analytes for all titration tests. The Stern–Volmer constant *K*_SV_ and the limit of detection LOD values (Table S8†) were calculated to be 3.16 × 10^4^ M^−1^ and 2.42 × 10^−7^ M 3.49 × 10^4^ M^−1^ and 2.19 × 10^−7^ M for IMI in the suspensions of Zn-1 and Zn-2, respectively.

### Recyclability experiments and fluorescence stability under different pH

**Fig. 9:**
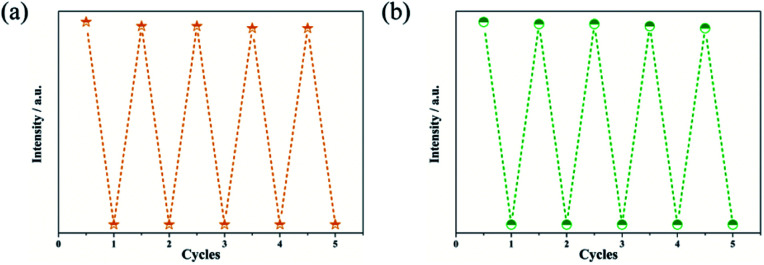
Recyclability of Zn-1 (a) and Zn-2 (b) implemented with 1 mM IMI.

Regeneration and recycling abilities are important features for fluorescent probes in feasible applications. In order to determine the reusability of the Zn-based complexes in sensing of analytes, recycling experiments were further studied. The complexes can be regenerated after a simple centrifugation and washes with deionized water. In addition, no obvious decrease in the initial intensity was observed even after 5 cycles of applications (Fig. 9 and S14†). Fluorescence stability of the sensors under different pH (range from 2 to 12) conditions was also checked. Fig. S15† displayed that there was an apex under the neutral condition. Ether decrease or increase of the pH of the suspension, a slightly decline of the fluorescence intensity of the sensor was observed.

### Discussion and possible mechanism for luminescent sensing

**Fig. 10:**
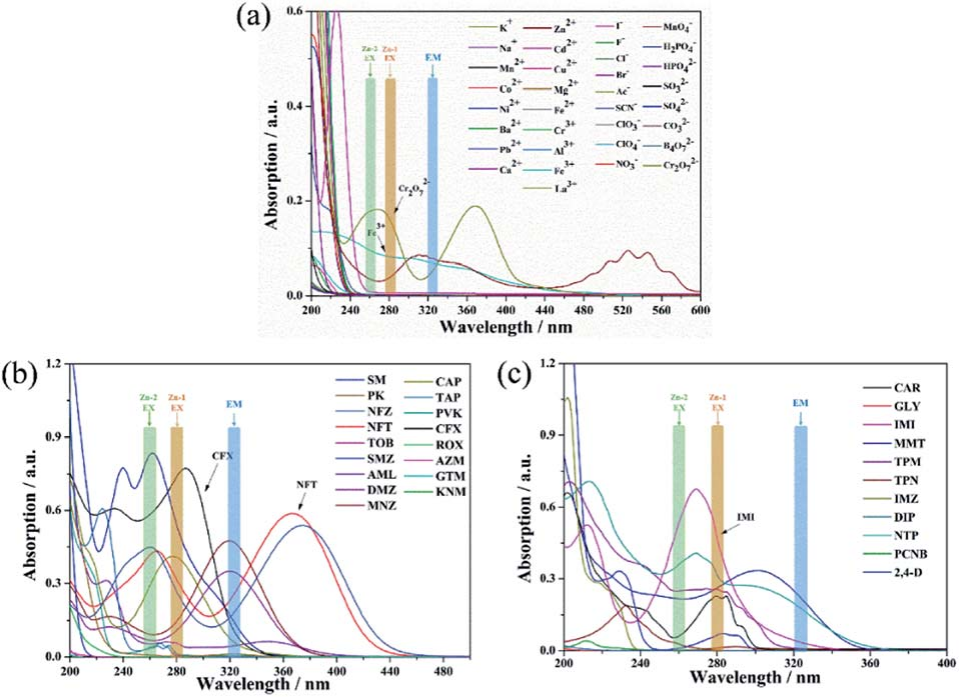
UV-vis absorption spectra of different ions (a), antibiotics (b) and pesticides (c).

**Fig. 11:**
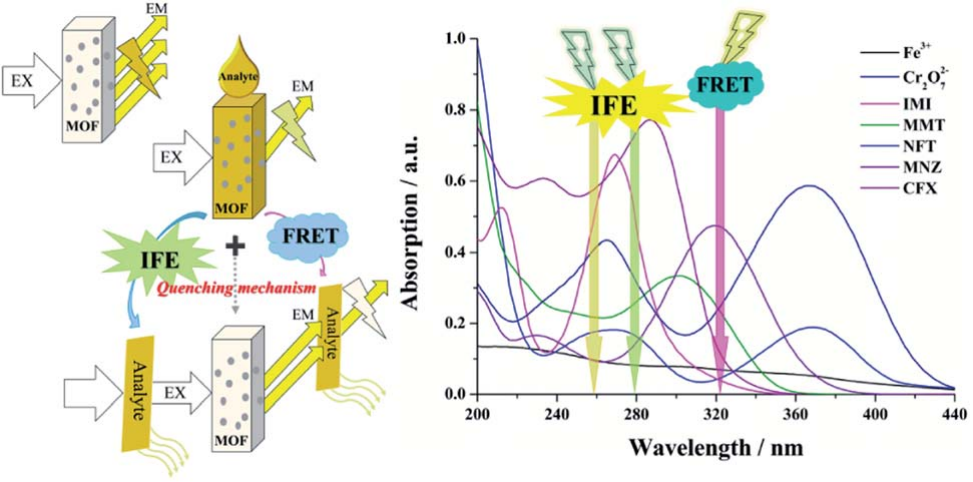
Schematic illustration of the fabrication route for the competition of incoming light by the Zn-MOFs and the analytes.

To better understand the intrinsic mechanism that is involved in the luminescent sensing process, a systematic investigation was performed and discussed. Generally, the effects on fluorescence can be ascribed to two major reasons: material transformation and energy transfer.^76^ From a structural point of view, skeletal eruption, weak or strong host and guest interactions and charge transfer are the main causes of fluorescence quenching.^59^ Taking this into account, FT-IR (Fig. S16†) and PXRD (Fig. S17†) analyses of mashed Zn complexes before and after immersion with various quenchers were recorded. No obvious differences were observed from the spectra before and after the fluorescence quenching progress, and the consistency of the spectra reveals that the fluorescence quenching is not caused by either abruption of the structure or strong binding of the quenchers and the sensors. Then, the search for the induction of fluorescence quenching moves to energy transfer. The energy-transfer mechanism includes the inner filter effect (IFE),^57^ fluorescence resonance energy transfer (FRET)^76^ and photoinduced electron transfer (PET) theory.^77^ FRET occurs through the transfer of energy from an excited donor to a ground state acceptor *via* a dipole–dipole interaction, which requires the wavelength of the emitted light to be located in the absorption band of the analytes.^76^ Therefore, the UV-vis absorption profiles of the analytes at a unique concentration in comparison with the excitation and emission of the sensors were evaluated and are exhibited in Fig. 10. There are clear overlaps between the emission band of the Zn complexes at 322 nm and those of analytes MnO_4_^−^, Fe^3+^ and Cr_2_O_7_^2−^ (Fig. 10a) CFX and NFT (Fig. 10b) and IMI (Fig. 10c). Therefore, the FRET mechanism was believed to play an important role in sensing these analytes. The IFE considers the competition for incoming light between the excitation of sensors and absorption of analytes.^57^Fig. 10 clearly shows that there are spectral overlaps between the absorption profiles of several antibiotics and pesticides and the emission curves of Zn complexes at 278 nm for Zn-1 and 259 nm for Zn-2. Referring back to Fig. 7, for Zn-1, the fluorescence quenching that was induced by the antibiotics is in the order of CFX > SMZ > NFT > NFZ > MNZ > DMZ > CAP > AML > the remaining antibiotics and for Zn-2, the antibiotics are ordered in terms of quenching rate as NFT > NFZ ≈ SMZ > MNZ ≈ CFX > DMZ > CAP > AML. Considering both the excitation and emission of the two Zn complexes and the absorption of the antibiotics, a sole mechanism could effectively explain neither the quenching rate nor their orders. The two mechanisms, which simultaneously involve fluorescence quenching, also occurred in the sensing progress of the tested pesticides and ions. Overall, in this study, both the IFE mechanism and FRET mechanism play important roles in sensing (Fig. 11). Moreover, all the obtained *K*_sv_ curves are linear at low quencher concentrations, thereby revealing dynamic quenching progress.^78,79^ With a higher concentration of analytes, the cures bend upwardly, thereby showing the involvement of static quenching.

## Conclusions

Two Zn-based complexes with 2D and 3D structural features were successfully synthesized and isolated with satisfactory purity. Their structural configurations and purities were analyzed and confirmed by using single crystal X-ray diffraction analysis, PXRD, IR, TGA and elemental analysis. Zn-1 adapts a 2-fold interpenetrated double-layered two-dimensional architecture. Zn-2 was built in a 4-nodal (3,4,4,5)-connected new topological 3D framework. The sensing capabilities of the Zn complexes were explored in cations, anions, antibiotics and pesticides. Fe^3+^, MnO_4_^−^ and Cr_2_O_7_^2−^ are selective and more sensitive than the other ions toward all two Zn complexes. In the progress of sensing antibiotics and pesticides, Zn-2 are extra sensitive toward NFT and IMI and Zn-1 performed outstandingly in sensing CFX and IMI. During sensing, both the IFE and FRET mechanisms play important roles in quenching the fluorescence intensity of the sensors.

## Conflicts of interest

There are no conflicts to declare.

## Supplementary Material

RA-012-D2RA00376G-s001

RA-012-D2RA00376G-s002
