# Supplementary material for: Two bis-ligand-coordinated Zn(ii)-MOFs for luminescent sensing of ions, antibiotics and pesticides in aqueous solutions
Source: RSC Adv. 2022 Mar 9;12(13):7780–8. doi: 10.1039/d2ra00376g (PMC8982467; doi:10.1039/d2ra00376g)
Supplement: RA-012-D2RA00376G-s001 [file RA-012-D2RA00376G-s001.pdf]

Electronic Supplementary Material (ESI) for RSC Advances.
This journal is © The Royal Society of Chemistry 2022

Two Bisligand-Coordinated Zn(II)-MOFs for Luminescent Sensing of Ions, Antibiotics and Pesticides in Aqueous Solutions

Zhao-Di Zhou, Shi-Qi Li, Yin Liu, Bin Du, Yuan-Yue Shen, Bao-Yi Yu* and Chong-Chen Wang*

Table S1 Crystal data for **Zn-1** and **Zn-2**

CCDC No.	2082187	2082188
Formula	$C_{54}H_{46}N_{28}O_{18}Zn_3$	$C_{33}H_{19}N_7O_8Zn_2$
Formula weight	1571.30	772.29
Temperature/K	150.0(1)	150.0(1)
Crystal system	triclinic	orthorhombic
Space group	P$\bar{1}$	$P2_12_12_1$
a / Å	10.1500(8)	7.6389(9)
b / Å	11.7115(10)	17.543(2)
c / Å	13.2760(12)	23.531(3)
α / °	81.779(3)	90.000(2)
β / °	72.548(2)	90
γ / °	87.653(3)	90.000(2)
V / Å^3	1490.0(2)	3153.4(7)
Z	1	4
ρ_{calc}[g/cm^3]	1.751	1.627
μ / mm^{-1}	1.300	1.587
F(000)	800.0	1560.0
Crystal size/mm^3	0.19×0.17×0.11	0.15 × 0.11 × 0.1
Radiation	MoKα(λ= 0.71073)	MoKα (λ = 0.71073)
2θ range for data	4.206 to 52.968	4.168 to 52.966
Index ranges	$-12 \le h \le 12$, $-14 \le k \le 14$, $-16 \le l \le 16$	$-9 \le h \le 9$, $-21 \le k \le 21$, $-29 \le l \le 29$
Reflections collected	17126	26810
Independent reflections	6096 [R_{int} = 0.0724, R_{sigma} = 0.0831]	6416 [R_{int} = 0.1640, R_{sigma} = 0.1649]
Data/restraints/parameters	6096/453/518	6416/780/534
Goodness-of-fit on F^2	1.063	1.035
Final *R* indexes [$I >= 2\sigma(I)$][a]	R_1 = 0.0646, wR_2 = 0.1644	R_1 = 0.1202, wR_2 = 0.2991
Final *R* indexes [all data][b]	R_1 = 0.1059, wR_2 = 0.1956	R_1 = 0.2132, wR_2 = 0.3545
Largest diff. peak/hole / e Å^{-3}	1.27/-0.72	1.91/-0.92

[a] $R_1 = \Sigma_1|F_0|-|F_C|/\Sigma_1|F_0|$; [b] $wR_2 = [\Sigma[w(F_0^2-F_C^2)^2]/\Sigma[(F_0^2)^2]]^{1/2}$.

Table S2 The selected bond lengths [Å] and angles [°] for **Zn-1**

Zn1O1W$^{\#1}$	2.137(4)	N9C19	1.307(8)	N1C10	1.320(7)	C1C2	1.501(7)
Zn1O1W	2.137(4)	N10C20	1.347(7)	N1C11	1.361(7)	C2C3	1.380(7)
Zn1O5	2.028(3)	N10C21	1.413(7)	N2N3	1.377(6)	C2C7	1.398(7)
Zn1O5$^{\#1}$	2.028(3)	N11C22	1.341(8)	N2C10	1.331(7)	C3C4	1.387(7)
Zn1N8	2.224(5)	N11C23	1.337(8)	N2C12	1.421(7)	C4C5	1.394(7)
Zn1N8$^{\#1}$	2.224(5)	N12C27	1.38(2)	N3C11	1.294(7)	C4C8	1.495(7)
Zn2O1	1.965(4)	N12C24	1.41(2)	N4C15	1.317(7)	C5C6	1.393(7)
Zn2O4$^{\#2}$	1.997(4)	N12N13	1.34(2)	N4C16	1.336(7)	C6C7	1.394(7)
Zn2N1	2.005(5)	N12AN13A	1.40(2)	N5N6	1.377(6)	C6C9	1.513(7)
Zn2N7$^{\#3}$	2.003(4)	N12AC24	1.49(2)	N5C14	1.421(7)	C12C13	1.382(7)
O1C1	1.281(6)	N12AC27A	1.33(2)	N5C17	1.331(7)	C12C16	1.383(8)
O2C1	1.243(6)	C27N14	1.30(2)	N6C18	1.309(7)	C13C14	1.378(8)
O3C8	1.250(6)	N13AC26A	1.34(2)	N7Zn2$^{\#5}$	2.003(4)	C14C15	1.385(8)
O4Zn2$^{\#4}$	1.997(3)	C26N13	1.38(2)	N7C17	1.322(7)	C21C22	1.376(8)
O4C8	1.281(6)	C26N14	1.30(2)	N7C18	1.352(7)	C21C25	1.405(8)
O5C9	1.266(6)	N14AC26A	1.28(2)	N8C19	1.365(7)	C23C24	1.372(8)
O6C9	1.244(6)	N14AC27A	1.35(2)	N8C20	1.315(7)	C24C25	1.383(8)
N9N10	1.374(6)						
O1WZn1O1W$^{\#1}$	180.0(2)	C26AN13AN12A	100(1)	N3N2C12	121.2(4)	C13C12C16	120.4(5)
O1W$^{\#1}$Zn1N8$^{\#1}$	87.1(2)	N14C26N13	103(1)	C10N2N3	109.7(4)	C16C12N2	119.8(5)
O1W$^{\#1}$Zn1N8	92.9(2)	C26AN14AC27A	105(1)	C10N2C12	129.0(4)	C14C13C12	116.6(5)
O1WZn1N8$^{\#1}$	92.9(2)	O1C1C2	117.2(4)	C11N3N2	102.4(4)	C13C14N5	120.0(5)
O1WZn1N8	87.1(2)	O2C1O1	122.4(5)	C15N4C16	117.9(5)	C13C14C15	119.8(5)
O5Zn1O1W$^{\#1}$	93(1)	O2C1C2	120.3(5)	N6N5C14	121.1(4)	C15C14N5	120.1(5)
O5$^{\#1}$Zn1O1W$^{\#1}$	87.0(1)	C3C2C1	118.7(4)	C17N5N6	110.0(4)	N4C15C14	123.2(6)
O5$^{\#1}$Zn1O1W	93(1)	C3C2C7	120.3(5)	C17N5C14	128.9(5)	N4C16C12	122.1(5)
O5Zn1O1W	87.0(1)	C7C2C1	120.9(5)	C18N6N5	102.1(4)	N7C17N5	109.4(5)
O5Zn1O5$^{\#1}$	180.0	C2C3C4	121.3(5)	C17N7Zn2$^{\#5}$	127.0(4)	N6C18N7	114.7(5)
O5Zn1N8$^{\#1}$	90.9(2)	C3C4C5	118.6(5)	C17N7C18	103.9(4)	N9C19N8	115.8(5)
O5Zn1N8	89.2(2)	C3C4C8	120.6(4)	C18N7Zn2^{5}	129.0(4)	N8C20N10	110.4(5)
O5$^{\#1}$Zn1N8	90.9(2)	C5C4C8	120.8(5)	C19N8Zn1	126.9(4)	C22C21N10	121.6(5)
O5$^{\#1}$Zn1N8$^{\#1}$	89.2(2)	C6C5C4	120.6(5)	C20N8Zn1	127.0(4)	C22C21C25	119.1(6)
N8Zn1N8$^{\#1}$	180.0(1)	C5C6C7	120.3(5)	C20N8C19	102.5(5)	C25C21N10	119.2(5)
O1Zn2O4$^{\#2}$	99.1(1)	C5C6C9	119.6(4)	C19N9N10	101.8(4)	N11C22C21	123.7(6)
O1Zn2N1	117(2)	C7C6C9	120.1(5)	N9N10C21	120.2(5)	N11C23C24	122.3(6)
O1Zn2N7$^{\#3}$	110.9(2)	C6C7C2	118.9(5)	C20N10N9	109.5(5)	C23C24N12	116.2(9)
O4$^{\#2}$Zn2N1	103.4(2)	O3C8O4	122.4(5)	C20N10C21	129.5(5)	C23C24N12A	123.8(9)
O4$^{\#2}$Zn2N7$^{\#3}$	107.4(2)	O3C8C4	119.8(4)	C23N11C22	117.4(5)	C23C24C25	121.3(6)
N7$^{\#3}$Zn2N1	116.7(2)	O4C8C4	117.8(4)	C27N12C24	119.4(13)	C25C24N12	121.3(9)
C1O1Zn2	105.0(3)	O5C9C6	115.6(4)	N13N12C27	114.0(14)	C25C24N12A	114.1(8)
C8O4Zn2$^{\#4}$	111.5(3)	O6C9O5	125.7(5)	N13N12C24	126.2(14)	C24C25C21	116.2(5)
C9O5Zn1	130.2(3)	O6C9C6	118.7(5)	N13AN12AC24	123(1)	N12N13C26	105(1)

C10N1Zn2	123.4(4)	N1C10N2	109.6(4)	C27AN12AN13A	110(1)	N14AC26AN13A	117(1)
C10N1C11	103.4(4)	N3C11N1	114.9(5)	C27AN12AC24	126(1)	C27N14C26	120(2)
C11N1Zn2	130.8(4)	C13C12N2	119.8(5)	N14C27N12	98(1)	N12AC27AN14A	109(1)

Symmetry transformation: $^{\#1}$1-X,1-Y,2-Z; $^{\#2}$-1+X,+Y,+Z; $^{\#3}$+X,1+Y,+Z; $^{\#4}$1+X,+Y,+Z; $^{\#5}$+X,-1+Y,+Z.

Table S3 The selected bond lengths [Å] and angles [°] for **Zn-2**

Zn1O1	2.03(2)	C27C28	1.41(3)	N2C25	1.33(3)	C22AC23A	1.3900
Zn1O8	2.07(2)	C28C29	1.35(3)	N3C28	1.38(3)	C9AC10A	1.46(7)
Zn1O7	2.02(2)	C17C18	1.43(3)	N3C26	1.49(3)	C9AC8A	1.31(7)
Zn1O2A	2.09(6)	C17C20	1.51(3)	N4C27$^{\#5}$	1.29(3)	C9C8	1.3900
Zn1O2	2.10(6)	C17C16	1.43(3)	N4C31$^{\#5}$	1.33(3)	C9C10	1.3900
Zn1N4	2.13(2)	C2C3	1.33(4)	N1C25	1.38(3)	C8C7	1.3900
Zn2O5	1.96(2)	C2C1	1.48(3)	N1C26	1.30(3)	C7C6	1.3900
Zn2O3	1.92(2)	C2C7	1.46(3)	N6N5	1.4200	C6C11	1.3900
Zn2O6	1.97(4)	C29C30	1.52(4)	N6C33	1.4200	C6C5	1.33(3)
Zn2O6A	1.98(4)	C3C4	1.45(4)	N5C32	1.4200	C11C10	1.3900
Zn2N1	1.98(2)	C3C8A	1.50(6)	N5C30	1.32(4)	C20O8$^{\#5}$	1.27(3)
O1C1	1.27(3)	C1O2A$^{\#7}$	1.26(7)	C32N7	1.4200	C20O7$^{\#8}$	1.34(3)
O8C20$^{\#1}$	1.27(3)	C1O2$^{\#7}$	1.21(7)	N7C33	1.4200	C12C5	1.50(3)
O5C13	1.25(3)	C11AC10A	1.27(7)	N1AN2A	1.4200	C15C16	1.3900
O7C20$^{\#2}$	1.34(3)	C11AC4	1.42(6)	N1AC32A	1.4200	C15C21	1.3900
O3C12	1.32(3)	C14C19	1.42(3)	N1AC30	1.42(5)	C16C24	1.3900
O6C13$^{\#3}$	1.29(5)	C14C15	1.42(3)	N2AC33A	1.4200	C24C23	1.3900
O2AC1$^{\#4}$	1.26(7)	C24AC18	1.3900	C33AN3A	1.4200	C23C22	1.3900
O4C12	1.19(3)	C24AC23A	1.3900	N3AC32A	1.4200	C22C21	1.3900
O2C1$^{\#4}$	1.21(7)	C18C19	1.3900	C13O6$^{\#6}$	1.29(5)	C4C5	1.36(4)
O6AC13^{3}	1.31(5)	C19C21A	1.3900	C13O6A$^{\#6}$	1.31(5)	C31N4$^{\#1}$	1.33(3)
N2N3	1.37(3)	C21AC22A	1.3900	C13C14	1.51(3)	C31C30	1.33(4)
C27N4$^{\#1}$	1.29(3)						
O1Zn1O8	90.6(6)	C16C17C20	117(2)	C1$^{\#4}$ O2 Zn1	148(6)	C7 C8 C9	120.0
O1Zn1O2A	177(2)	C3C2C1	123(2)	C13$^{\#3}$O6AZn2	129(3)	C8 C7 C2	125(2)
O1Zn1O2	173(2)	C3C2C7	119(2)	C25 N2 N3	103(2)	C8 C7 C6	120.0
O1Zn1N4	92.0(7)	C7C2C1	118(2)	N2 N3 C28	121(2)	C6 C7 C2	115(2)
O8Zn1O2A	93(2)	N1C26N3	105(2)	N2 N3 C26	109(2)	C11 C6 C7	120.0
O8Zn1O2	83(2)	C28C29C30	112(2)	C28 N3 C26	129(2)	C5 C6 C7	126(2)
O8Zn1N4	145.9(7)	C2C3C4	120(3)	C27$^{\#5}$ N4 Zn1	119(2)	C5 C6 C11	114(2)
O7Zn1O1	92.4(6)	C2C3C8A	124(3)	C27$^{\#5}$N4C315	120(2)	C6C11C10	120.0
O7Zn1O8	104.1(6)	C4C3C8A	114(3)	C31$^{\#5}$N4Zn1	121(2)	C11C10C9	120.0
O7Zn1O2A	87(2)	O1C1C2	120(2)	C25N1Zn2	127(2)	O8$^{\#5}$C20O7$^{\#8}$	121(2)
O7Zn1O2	90(2)	O2A$^{\#7}$C1O1	126(4)	C26N1Zn2	124(2)	O8$^{\#5}$C20C17	119(2)
O7Zn1N4	109.8(7)	O2A$^{\#7}$C1C2	115(4)	C26N1C25	108(2)	O7$^{\#8}$C20C17	120(2)
O2AZn1O2	10(3)	O2$^{\#7}$C1O1	121(4)	N5N6C33	108.0	O3C12C5	116(2)
O2AZn1N4	85(2)	O2$^{\#7}$C1O2A$^{\#7}$	17(5)	C32N5N6	108.0	O4C12O3	123(2)
O2Zn1N4	93(2)	O2$^{\#7}$C1C2	117(4)	C30N5N6	125(4)	O4C12C5	121(3)

O5Zn2O6	105(2)	C10AC11A C4	126(5)	C30N5C32	127(4)	C16C15C14	116(2)
O5Zn2O6A	98(1)	C19C14C13	118(2)	N5C32N7	108.0	C16C15C21	120.0
O5Zn2N1	117.7(8)	C15C14C13	116(2)	C32N7C33	108.0	C21C15C14	124(2)
O3Zn2O5	108.7(8)	C15C14C19	126(2)	N7C33N6	108.0	C15C16C17	119(2)
O3Zn2O6	106(1)	C18C24AC23A	120.0	N2AN1AC32A	108.0	C24C16C17	122(2)
O3Zn2O6A	120(1)	C24AC18C17	124(2)	N2AN1AC30	126(3)	C24C16C15	120.0
O3Zn2N1	104.3(8)	C19C18C17	116(2)	C32AN1AC30	121(3)	C16C24C23	120.0
O5Zn2O6	105(2)	C10AC11AC4	126(5)	C33AN2AN1A	108.0	C22C23C24	120.0
O5Zn2O6A	98(1)	C19C14C13	118(2)	N3AC33AN2A	108.0	C23C22C21	120.0
O5Zn2N1	117.7(8)	C15C14C13	116(2)	C32AN3AC33 A	108.0	C22C21C15	120.0
O3Zn2O5	108.7(8)	C15C14C19	126(2)	N3AC32AN1A	108.0	C11AC4C3	117(3)
O3Zn2O6	106(1)	C18C24AC23A	120.0	O5C13O6#6	120(3)	C5C4C3	120(3)
O3Zn2O6A	120(1)	C24AC18C17	124(2)	O5C13O6A#6	119(3)	C5C4C11A	121(3)
O3Zn2N1	104.3(8)	C19C18C17	116(2)	O5C13C14	115(2)	C30C31N4#1	122(3)
O6Zn2O6A	14(2)	C19C18C24A	120.0	O6#6C13O6A#6	21(2)	C6C5C12	120(2)
O6Zn2N1	114(2)	C18C19C14	118(2)	O6#6C13C14	124(3)	C6C5C4	118(2)
O6AZn2N1	109(1)	C18C19C21A	120.0	O6A#6C13C14	124(3)	C4C5C12	122(2)
C1O1Zn1	133(2)	C21AC1914	121(2)	N2C25N1	115(2)	N5C30C29	122(3)
C20#1O8Zn1	104(2)	C22AC21AC19	120.0	N4#1C27C28	122(2)	N5C30C31	116(3)
C13O5Zn2	121(2)	C21AC22AC23 A	120.0	N3C28C27	118(2)	N1AC30C29	111(3)
C20#2O7Zn1	120(1)	C22AC23AC24 A	120.0	C29C28N3	119(2)	C31C30N1A	127(3)
C12O3Zn2	113(2)	C8AC9AC10A	120(5)	C29C28C27	122(2)	C31C30C29	120(3)
C13#3O6Zn2	131(3)	C11AC10AC9A	119(5)	C18C17C20	118(2)	C9AC8AC3	122(5)
C1#4O2AZn1	143(5)	C8C9C10	120.0	C16C17C18	125(2)		

Symmetry transformation: #1 1-X,-1/2+Y,1/2-Z; #2 1/2-X,2-Y,-1/2+Z; #3 -1/2+X,3/2-Y,1-Z; #4 -1/2+X,3/2-Y,-Z; #5 1-X,1/2+Y,1/2-Z; #6 1/2+X,3/2-Y,1-Z; #7 1/2+X,3/2-Y,-Z; #8 1/2-X,2-Y,1/2+Z.

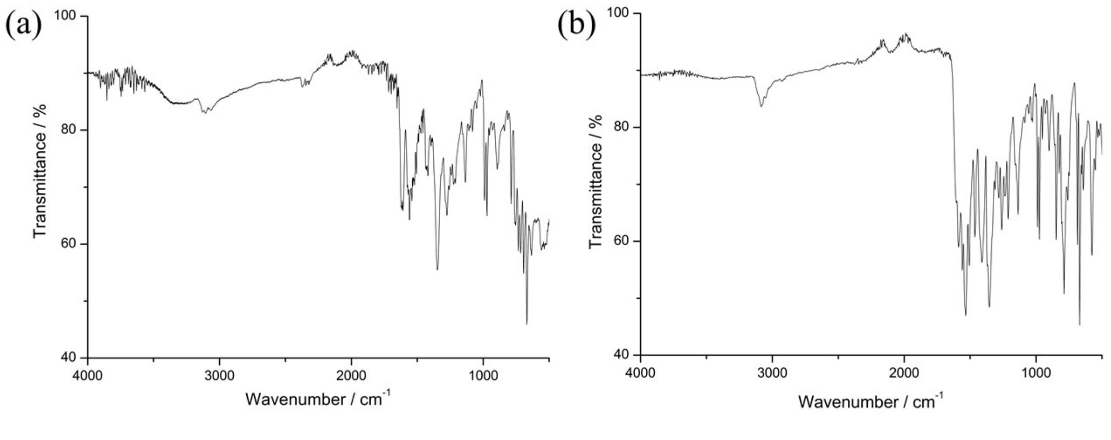

Fig. S1 IR spectra of **Zn-1** (a) and **Zn-2** (b) .

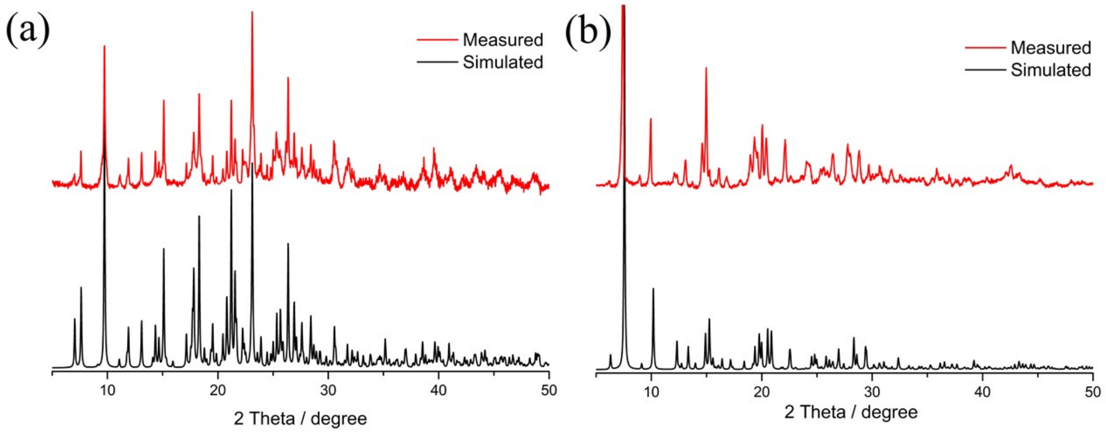

Fig. S2 PXRD profiles for **Zn-1** and **Zn-2**. Simulated spectrum was calculated from the single crystal data.

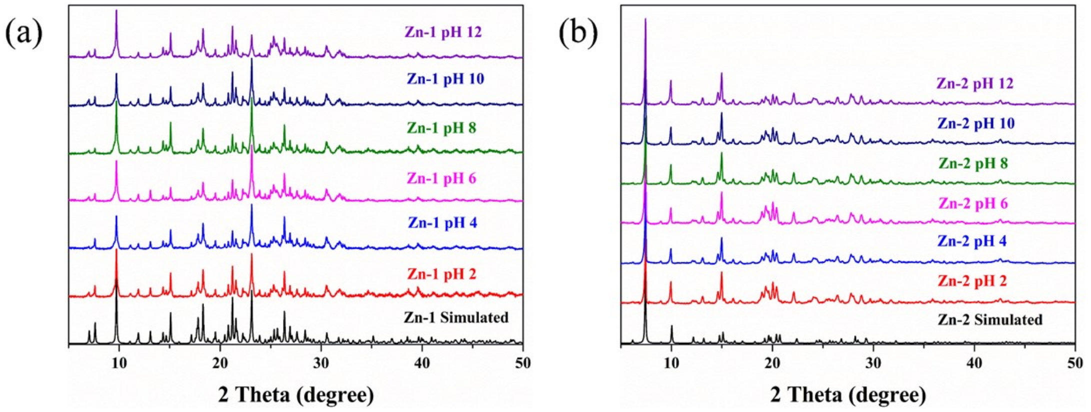

Fig. S3 PXRD patterns of **Zn-1** (a) and **Zn-2** (b) in an aqueous suspensions with the pH value ranging from 2 to 12.

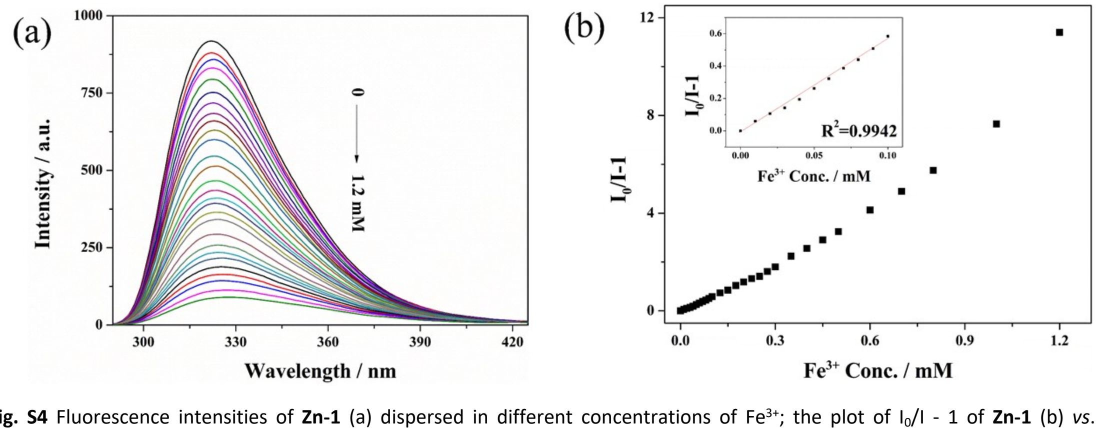

Fig. S4 Fluorescence intensities of **Zn-1** (a) dispersed in different concentrations of Fe^{3+}; the plot of I_0/I - 1 of **Zn-1** (b) *vs*. concentration of Fe^{3+} in aqueous solution (Insert: The plot of I_0/I - 1 of the MOFs with the concentration over a Fe^{3+} concentration range of 0-0.1 mM in aqueous solution).

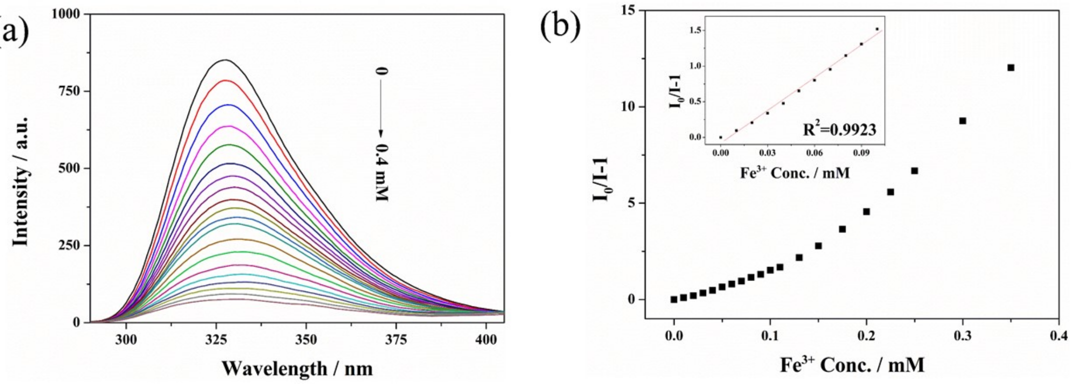

Fig. S5 Fluorescence intensities of **Zn-2** (a) dispersed in different concentrations of Fe^{3+}; the plot of I_0/I - 1 of **Zn-2** (b) *vs*. concentration of Fe^{3+} in aqueous solution (Insert: The plot of I_0/I - 1 of the MOFs with the concentration over a Fe^{3+} concentration range of 0-0.1 mM in aqueous solution).

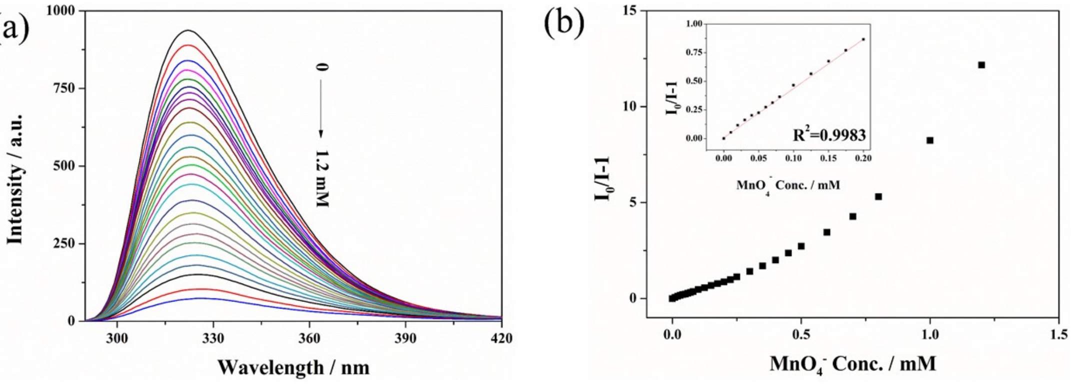

Fig. S6 Fluorescence intensities of **Zn-1** (a) dispersed in different concentrations of MnO_4^-; the plot of I_0/I - 1 of **Zn-1** (b) *vs*. concentration of MnO_4^- in aqueous solution (Insert: The plot of I_0/I - 1 of the MOF with the concentration over a MnO_4^- concentration range of 0 - 0.2 mM in aqueous solution).

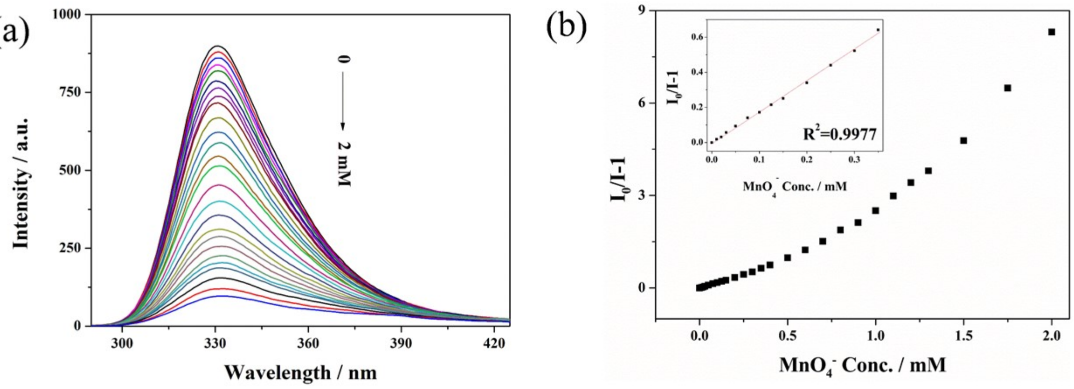

Fig. S7 Fluorescence intensities of **Zn-2** (a) dispersed in different concentrations of MnO_4^-; The plot of I_0/I - 1 of **Zn-2** (b) *vs*. concentration of MnO_4^- in aqueous solution (Insert: The plot of I_0/I - 1 of the MOF with the concentration over a MnO_4^- concentration range of 0 - 0.35 mM in aqueous solution).

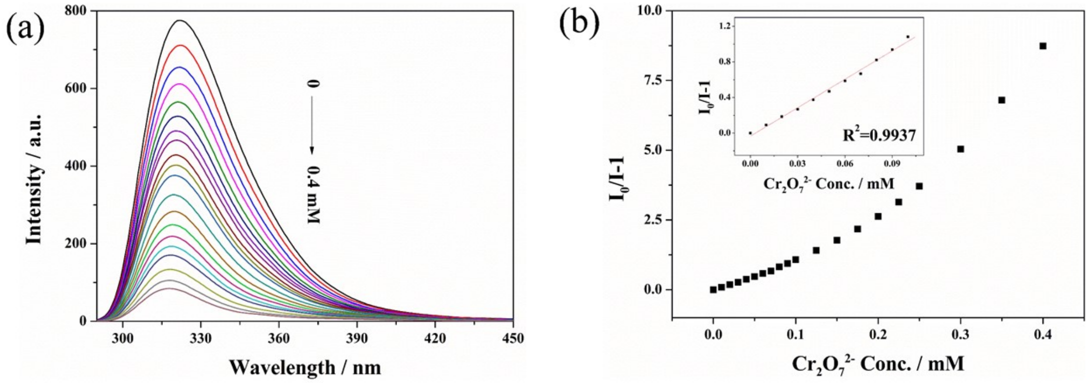

Fig. S8 Fluorescence intensities of **Zn-1** (a) dispersed in different concentrations of $Cr_2O_7^{2-}$; the plot of I_0/I - 1 of **Zn-1** (b) *vs*. concentration of $Cr_2O_7^{2-}$ in aqueous solution (Insert: The plot of I_0/I - 1 of the MOFs with the concentration over a $Cr_2O_7^{2-}$ concentration range of 0-0.1 mM in aqueous solution).

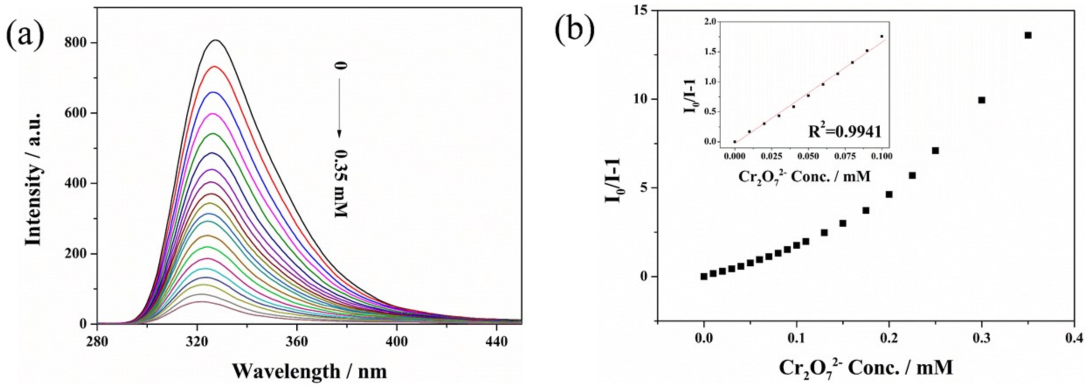

Fig. S9 Fluorescence intensities of **Zn-2** (a) dispersed in different concentrations of $Cr_2O_7^{2-}$; the plot of I_0/I - 1 of **Zn-2** (b) *vs*. concentration of $Cr_2O_7^{2-}$ in aqueous solution (Insert: The plot of I_0/I - 1 of the MOFs with the concentration over a $Cr_2O_7^{2-}$ concentration range of 0-0.1 mM in aqueous solution).

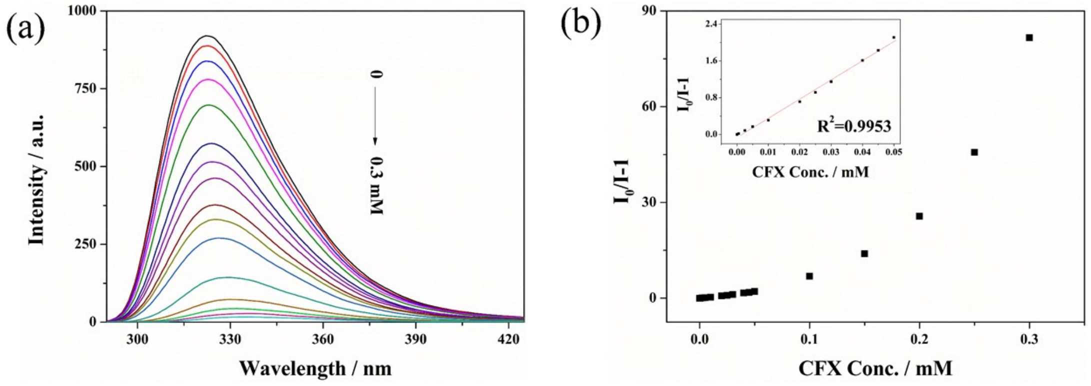

Fig. S10 Fluorescence intensities of **Zn-1** (a) dispersed in different concentrations of CFX; the plot of I_0/I - 1 of **Zn-1** (b) *vs*. concentration of CFX in aqueous solution (Insert: The plot of I_0/I - 1 of the MOF with the concentration over a CFX concentration range of 0 - 0.05 mM in aqueous solution).

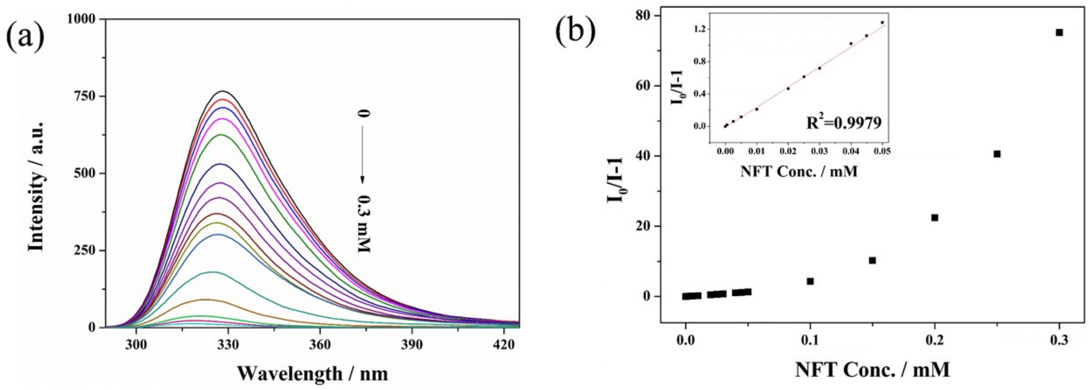

Fig. S11 Fluorescence intensities of **Zn-2** (a) dispersed in different concentrations of NFT; the plot of I_0/I - 1 of **Zn-2** (b) *vs*. concentration of NFT in aqueous solution (Insert: The plot of I_0/I - 1 of the MOF with the concentration over a NFT concentration range of 0 - 0.05 mM in aqueous solution).

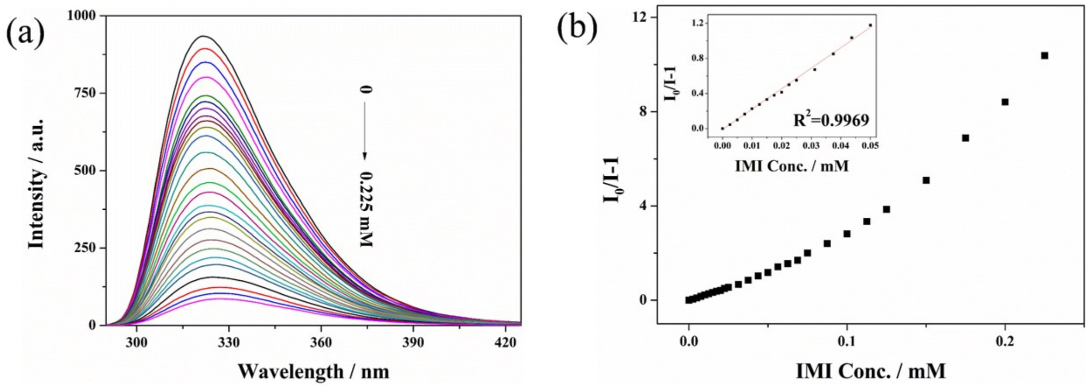

Fig. S12 Fluorescence intensities of **Zn-1** (a) dispersed in different concentrations of IMI; the plot of I_0/I - 1 of **Zn-1** (b) *vs*. concentration of IMI in aqueous solution (Insert: The plot of I_0/I - 1 of the MOF with the concentration over a IMI concentration range of 0 - 0.05 mM in aqueous solution).

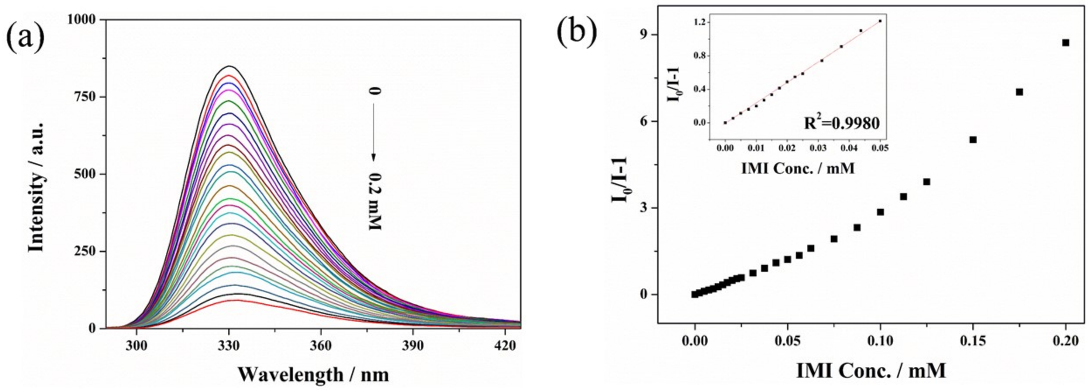

Fig. S13 Fluorescence intensities of **Zn-2** (a) dispersed in different concentrations of IMI; the plot of I_0/I - 1 of **Zn-2** (b) *vs*. concentration of IMI in aqueous solution (Insert: The plot of I_0/I - 1 of the MOF with the concentration over a IMI concentration range of 0 - 0.05 mM in aqueous solution).

Table S4 Ksv and LOD values of **Zn-1** and **Zn-2** for the ions detection

Sensor	Analyte	Concentration / mg·mL^{-1}	Linear range / μM	LOD / μM	Ksv × 10^4 / M^{-1}
**Zn-1**	Fe^{3+}	0.1	0-100	1.10	0.70
**Zn-2**	Fe^{3+}	0.1	0-100	0.50	1.53
**Zn-1**	MnO_4^-	0.1	0-200	3.32	0.23

**Zn-2**	MnO_4^-	0.1	0-350	4.30	0.18
**Zn-3**	$Cr_2O_7^{2-}$	0.1	0-100	0.72	1.07
**Zn-2**	$Cr_2O_7^{2-}$	0.1	0-100	0.44	1.73

Table S5 Chemical structure of the selected antibiotics

Kind	Name (abbreviation)	Chemical structure
Lactams	Benzylpenicillin potassium (PK)	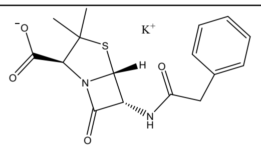
	Penicillin V potassium (PVK)	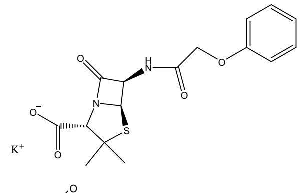
	Amoxicillin (AML)	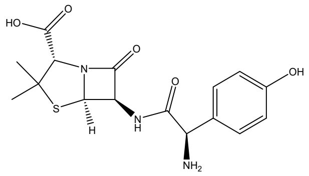
	Cefixime (CFX)	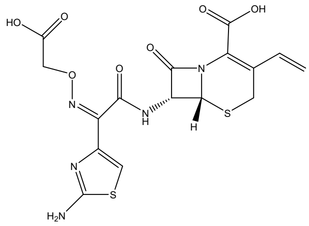
Aminoglycosides	Gentamicin (GTM)	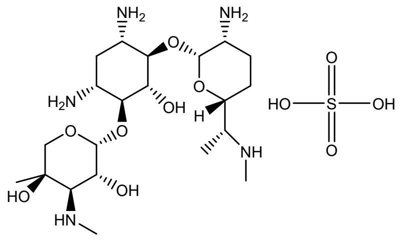
	Kanamycin (KNM)	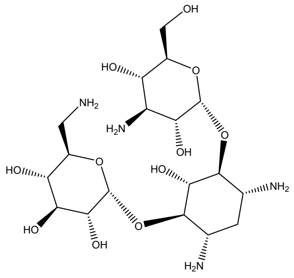
	Tobramycin (TOB)	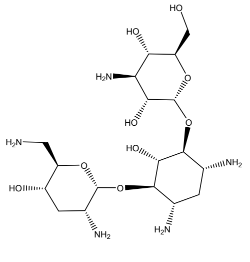

	Streptomycin (SM)	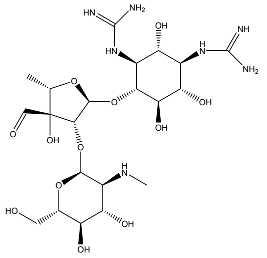
Chloramphenicols	Chloramphenicol (CAP)	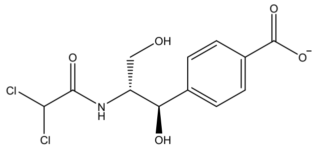
	Thiamphenicol (TAP)	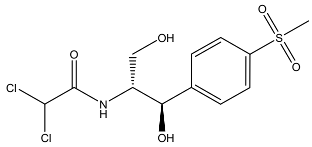
Macrolides	Roxithromycin (ROX)	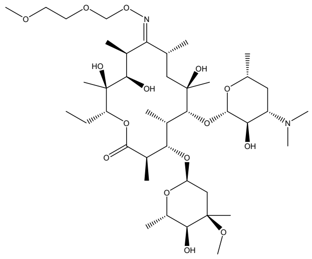
	Azithromycin (AZM)	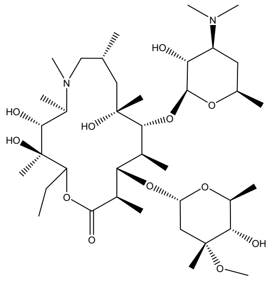
Nitrofurans	Nitrofurantoin (NFT)	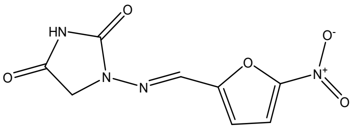
	Nitrofurazone (NFZ)	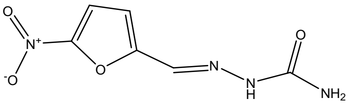
Nitroimidazoles	Metronidazole (MNZ)	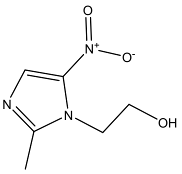
	1,2-dimethyl-5-nitroimidazole (DMZ)	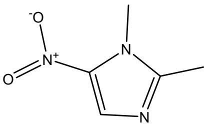
Sulfonamides	Sulfamethazine (SMZ)	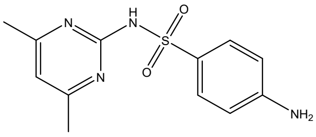

Table S6 Chemical structure of the selected pesticides

Name (abbreviation)	Chemical structure
Dipterex (DIP)	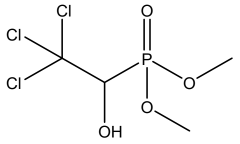
Pentachloro-nitrobenzene (PCNB)	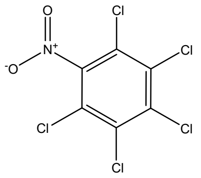
Imazalil (IMZ)	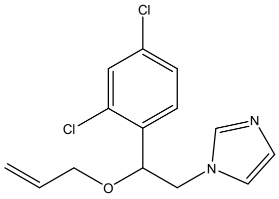
Glyphosate (GLY)	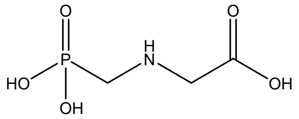
Chlorothalonil (TPN)	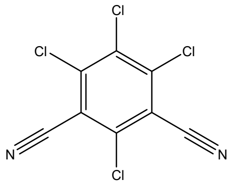
Carbendazim (CAR)	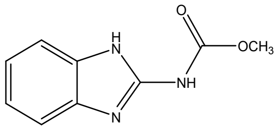
2.4-dichlorophenoxyacetic acid (2,4-D)	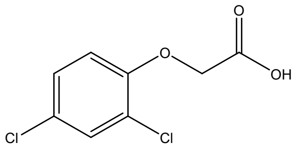
Imidacloprid (IMI)	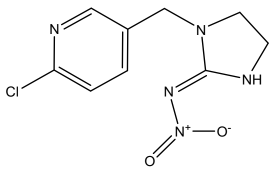
Metamitron (MMT)	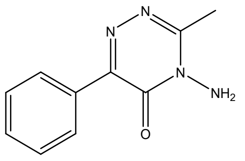
Thiophanate-methyl (TPM)	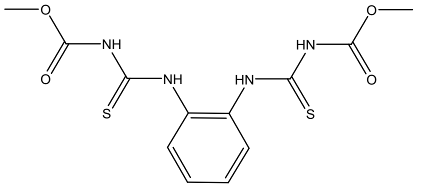

Nitenpyram (NTP)	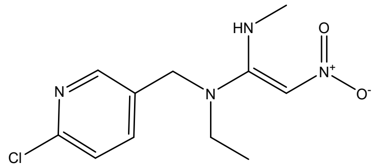

Table S7 Ksv and LOD values for recently reported lanthanide MOF-based luminescence probes for sensing of NFT and CFX

Sensor	Analyte	Concentration / mg·mL^{-1}	Linear range / μM	LOD / μM	Ksv × 10^4 / M^{-1}	Ref.
**Zn-1**		0.1	0-50	0.18	4.14	This work
Zn-PDC/Tb^{3+}	CFX	1.0	0-10	0.14	11	[1]
RhB@Tb-dcpcpt		1.0	0-100	1.579	1.67	[2]
Cd-MOF/Tb^{3+}		0.1	0-6.5	0.0267	6.16	[3]
MIP@TbMOF-76		-	1.76-198	48.5	-	[4]
**Zn-2**		0.1	0-50	0.28	2.74	This work
$\$[Zn(TTPBA\text{-}4)_{0.5}(TPA)]\cdot H_2O\cdot 0.5DMF\}_n$		-	0-10	-	2.0	[5]
$\$[Zn(L)]\cdot CH_3CN\}_n$		0.6	-	3.28	248	[6]
RhB@ZIF-8		0.8	0-38	0.47	1.8	[7]
FSS@ZIF-8	NFT	0.8	0-38	0.35	2.0	[8]
$\$[Zn_2(TRZ)_2(DBTDC\text{-}O_2)]\cdot DMAc\}_n$		1.0	4-20	0.353	18	[9]
$[(Zn_4O)_2(PDDA)_6(H_2O)_2]\cdot 10DMF$		0.1	0-50	-	11.6	[10]
$[Zn_8(C_5H_4N_5)_4(C_{14}H_8O_4)_6O(C_{50}H_{44}N_4)_{0.5}]$		0.5	-	0.563	4.42	[11]
$\$[Zn(tptc)_{0.5}(bimb)]\cdot H_2O\}_n$		0.5	-	0.49	4.51	[12]
$[Zn(DCPP)(H_2O)]\cdot(DMF)$		0.33	0-120	0.14	6.42	[13]

Table S8 Ksv and LOD values for recently reported MOF-based luminescence probes for IMI

Sensor	Analyte	Concentration / mg·mL^{-1}	Linear range / μM	LOD / μM	Ksv × 10^4 / M^{-1}	Ref.
**Zn-1**	IMI	0.1	0-50	0.35	2.16	This work
**Zn-2**	IMI	0.1	0-50	0.31	2.49	This work

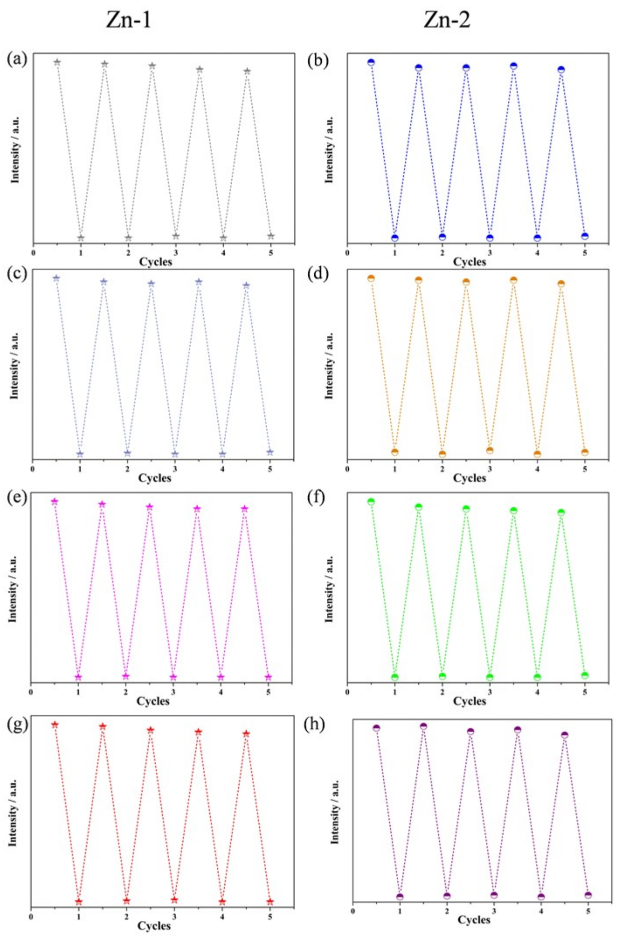

Fig. S14 Recyclability of the CPs implemented with 1 mM of the analytes (**Zn-1**: (a) Fe^{3+}, (c) MnO_4^-, (e) $Cr_2O_7^{2-}$, (g) CFX and **Zn-2**: (b) Fe^{3+}, (d) MnO_4^-, (f) $Cr_2O_7^{2-}$, (h) NFT) in aqueous solution.

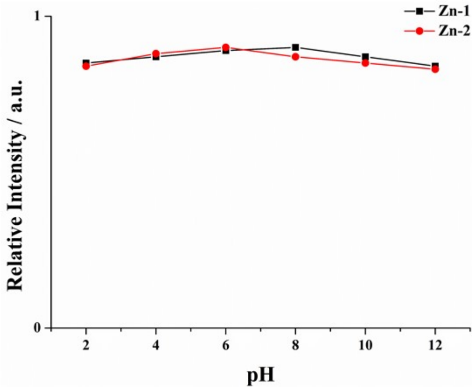

Fig. S15 The fluorescence intensity of **Zn-1** and **Zn-2** under different pHs.

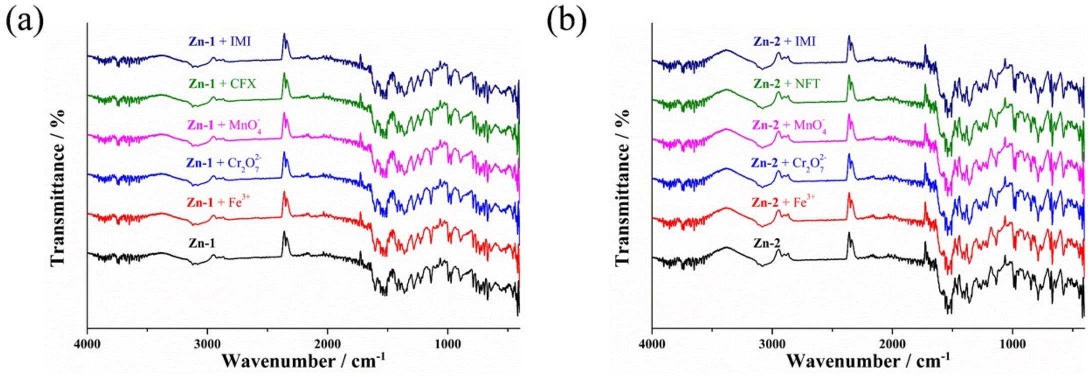

Fig. S16 Comparison before and after adding test substance of FT-IR spectra of **Zn-1** (a) and **Zn-2** (b).

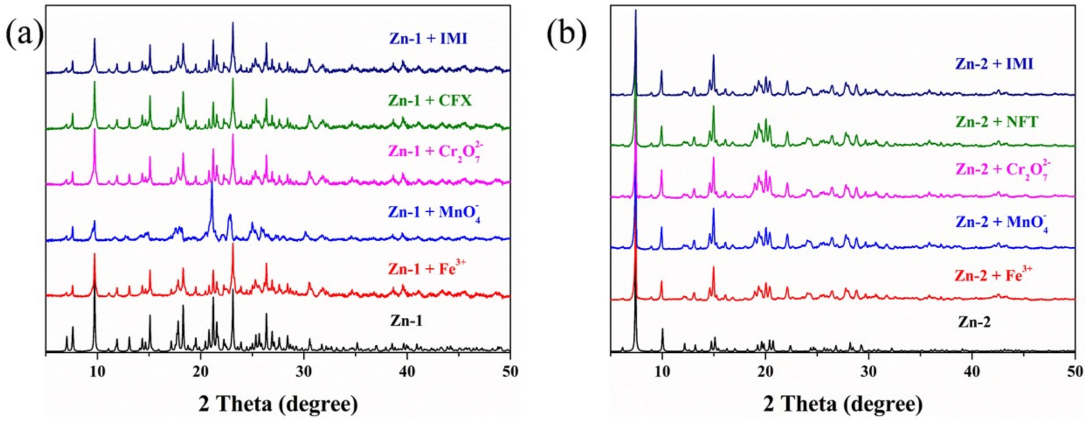

Fig. S17 Comparison before and after adding test substance of p-XRD patterns of **Zn-1** (a) and **Zn-2** (b).

References

1. H. Pan, S. Wang, X. Dao and Y. Ni, *Inorg. Chem.*, **2018**, 57 (3), 1417-1425.
2. M. Yu, Y. Xie, X. Wang, Y. Li and G. Li, *ACS Appl. Mater. Inter.*, **2019**, 11 (23), 21201-21210.
3. G. Qin, J. Wang, L. Li, F. Yuan, Q. Zha, W. Bai and Y. Ni, *Talanta*, **2021**, 221, 121421.
4. H. Eskandari, M. Amirzehni, H. Asadollahzadeh, J. Hassanzadeh and P. A. Eslami, *Sens. Actuators, B. Chem.*, **2018**, 275, 145-154.
5. H.B. Zhu, Y. Shen, Z.Z. Fu, Y.Y. Yu, Y.F. Jiang and Y. Zhao, *Inorg. Chem. Commun.*, **2019**, 103, 21-24.
6. J. Li, T.J. Chen, S. Han and L.F. Song, *J. Solid State Chem.*, **2019**, 277, 107-114.
7. Y. Q. Zhang, X. H. Wu, S. Mao, W. Q. Tao and Z. Li, *Talanta*, **2019**, 204, 344-352.
8. H. He, Q.Q. Zhu, C.P. Li and M. Du, *Cryst. Growth Des.*, **2018**, 19 (2), 694-703.
9. H. He, Q.Q. Zhu, M.T. Guo, Q.S. Zhou, J. Chen, C.P. Li and M. Du, *Cryst. Growth Des.*, **2019**, 19 (9), 5228-5236.
10. Y.M. Ying, C.L. Tao, M. Yu, Y. Xiong, C.R. Guo, X.G. Liu and Z. Zhao, *J. Mater. Chem. C*, **2019**, 7 (27), 8383-8388.
11. L. Fan, F. Wang, D. Zhao, Y. Peng, Y. Deng, Y. Luo and X. Zhang, *Appl. Organomet. Chem.,* **2020**, 34 (12), e5960.
12. S.L. Sun, X.Y. Sun, Q. Sun and E.Q. Gao, *CrystEngComm*, **2021**, 23 (17), 3167-3174.
13. J. Wang, Q. Zha, G. Qin and Y. Ni, *Talanta*, **2020**, 211, 120742.
